# EDEM1 Inhibits Endoplasmic Reticulum Stress to Induce Doxorubicin Resistance through Accelerating ERAD and Activating Keap1/Nrf2 Antioxidant Pathway in Triple-Negative Breast Cancer

**DOI:** 10.34133/research.0797

**Published:** 2025-07-29

**Authors:** Yajie Wang, Yiran Liang, Dan Luo, Fangzhou Ye, Yuhan Jin, Lei Wang, Yaming Li, Dianwen Han, Zekun Wang, Bing Chen, Wenjing Zhao, Lijuan Wang, Qifeng Yang

**Affiliations:** ^1^ Department of Breast Surgery, General Surgery, Qilu Hospital of Shandong University, Jinan, Shandong 250012, China.; ^2^ Biological Resource Center, Qilu Hospital of Shandong University, Jinan, Shandong 250012, China.; ^3^ Research Institute of Breast Cancer, Shandong University, Jinan, Shandong 250012, China.

## Abstract

Doxorubicin (DOX)-based chemotherapy is the basic treatment for triple-negative breast cancer (TNBC). However, chemoresistance is still one of the major causes of metastasis, recurrence, and poor outcomes. Recently, a close relationship between chemoresistance and endoplasmic reticulum (ER) stress has been found. In this study, ER-associated degradation (ERAD)-related protein EDEM1 (ER degradation enhancing α-mannosidase-like 1) plays a vital role in DOX-induced ER stress, which is up-regulated in tumor cells and tissues. In vitro and in vivo experiments reveal the promoting role of EDEM1 in the progression and chemoresistance of TNBC. Besides, EDEM1 attenuates autophagy and reduces ER stress-related apoptosis, indicating its inhibitory effect on ER stress. Furthermore, EDEM1 promotes ERAD and enhances the antioxidant capacity of tumor cells. Mechanistically, EDEM1 competitively binds Kelch-like ECH-associated protein 1 to prevent the ubiquitination and degradation of nuclear factor erythroid 2-related factor 2 (Nrf2), leading to increased Nrf2 nuclear translocation and antioxidant response element activation to bolster antioxidant defense and cell survival. Moreover, both the expression and function of EDEM1 are down-regulated by miR-32-5p. Clinically, high EDEM1 expression is correlated with poor patient outcomes in breast cancer, especially in TNBC patients treated with DOX-based chemotherapy. These findings reveal EDEM1 as a regulator of ER homeostasis during cancer progression and chemoresistance, and a potential target for breast cancer therapy.

## Introduction

Breast cancer is the most commonly diagnosed cancer and the second leading cause of cancer-related deaths among women worldwide [1]. Triple-negative breast cancer (TNBC) is a subtype defined by lacking estrogen receptor, progesterone receptor expression, and human epidermal growth factor receptor 2 (HER2) overexpression/amplification [2], which constitutes 10% to 20% of all breast cancers [3]. Compared to the other subtypes, TNBC has a more highly aggressive clinical course, earlier onset age, greater metastatic potential, higher relapse, lower survival rates, and ultimately poorer clinical outcomes [4–6]. Due to the lack of effective therapeutic targets, TNBC patients cannot benefit from endocrine therapy and anti-HER2 therapy, and chemotherapy is the principal therapeutic option in TNBC. The preferred regimens include taxanes and anthracyclines, and platinum-based regimens are also used in neoadjuvant therapy and metastatic disease [7]. Doxorubicin (DOX), an anthracycline class of cytotoxic chemotherapeutic drugs, is the standard of care treatment for TNBC [8]. However, the existence of chemoresistance markedly reduced the remission rate to 20% to 30% [9], especially in the metastatic setting where it accounts for 90% of therapy failure [10], leading to cancer progression and poor prognosis of TNBC. Nonetheless, the mechanisms of how TNBC cells develop chemoresistance to DOX are complicated and need further exploration. Therefore, elucidating the mechanism of chemoresistance to DOX is of great significance for discovering novel therapeutic targets and improving patient prognosis of TNBC.

The endoplasmic reticulum (ER), an important organelle for protein synthesis, modifications, and transport, is exquisitely sensitive to changes in the environment [11]. Intrinsic and extrinsic cellular stresses, such as nutrient deprivation, hypoxia, pH alterations, redox state, and calcium balance disorder, could lead to the accumulation of unfolded or misfolded protein and induce ER stress [11,12]. Previous studies reported that ER stress was involved in various biological processes, disease progression, and chemoresistance of multiple cancers [13–15], serving as a ubiquitous defense mechanism to confront stimuli through activating downstream signaling pathways. However, enduring or intense ER stress could also cause damage to other organelles and even cell death [16]. To maintain ER homeostasis, cells have evolved an ER quality control (ERQC) system to promote protein folding and eliminate misfolded proteins through 3 pathways: unfolded protein response (UPR), ER-associated degradation (ERAD), and autophagy [17]. UPR was mainly mediated by 3 branches, including protein kinase RNA-like ER kinase (PERK), activating transcription factor 6 alpha (ATF6α), and inositol-requiring kinase 1 (IRE1) [18], which could promote protein folding or reduce protein translation to prevent further protein accumulation in ER. On the other hand, ERAD and autophagy help to degrade misfolded proteins and aggregated proteins, while degradation failure further leads to the trigger of UPR [19]. In addition, redox regulation of ER is delicate and sensitive to perturbation, the antioxidant defense system also plays an important role in coping with the up-regulation of ROS after ER stress [20]. These adaptive mechanisms not only play a vital role in normal physiological conditions but also help cancer cells adapt to harsh conditions such as chemotherapy [21]. It was reported that DOX could up-regulate ROS production, thus affecting the protein folding ability in ER and causing ER stress [22,23]. However, the detailed mechanism involved in the defense of DOX-induced ER stress in breast cancer has not been fully elucidated.

ERAD involves 3 steps: recognition of improperly folded or formless proteins in the ER; retro-translocation into the cytosol; and ubiquitin-dependent degradation by the proteasome [24]. ER degradation enhancing α-mannosidase-like (EDEM) family of mannose-processing proteins has been shown to catalyze mannose trimming for glycans exposed on partially folded or misfolded glycoproteins, thus accelerating their degradations, which includes 3 family members: EDEM1, EDEM2, and EDEM3 [25]. EDEM1, an ER-resident protein, showed a crucial role in extracting misfolded polypeptides from the calnexin cycle as the first step in ERAD [26]. Moreover, the N-terminal disordered region of EDEM1 mediates protein–protein interaction with the misfolded proteins. In addition, when proteasomal activity is severely impaired, EDEM1 can still induce degradation by promoting the formation of aggregates and activating autophagy [27]. A few studies have shown that the EDEM family members are associated with tumor progression while the underlying mechanism is not clear. For example, EDEM1 was related to cancer progression and poor prognosis in lung adenocarcinoma and colorectal cancer [28,29]. EDEM2 was associated with immune infiltration and can be a diagnostic and prognostic biomarker of glioma [30]. EDEM3 promoted radioresistance in prostate cancer cells [31]. However, the role of EDEM1 in the progression and drug resistance of TNBC has not been reported.

In this study, we found that the level of EDEM1 was induced upon ER stress and DOX treatment in TNBC cells. Also, EDEM1 was found to be closely related to TNBC progression and DOX resistance through accelerating ERAD and attenuating oxidative stress. Moreover, EDEM1 relieved ER stress by competitively binding to the double glycine repeat (DGR) domain of Kelch-like ECH-associated protein 1 (Keap1) with nuclear factor erythroid 2-related factor 2 (Nrf2), reducing the ubiquitination and degradation of Nrf2, finally activating antioxidant response element (ARE) and downstream antioxidant molecules to reduce ROS levels during DOX-induced ER stress. Besides, the expression and function of EDEM1 were negatively regulated by upstream miR-32-5p. Furthermore, the expressions of EDEM1 were associated with worse clinical outcomes in breast cancer patients. Our study not only demonstrated the crucial role of EDEM1 in DOX-induced ER stress but also provided a potential therapeutic target and prognostic predictor for breast cancer patients.

## Results

### EDEM1 is involved in DOX-induced ER stress in TNBC

**Fig. 1.**
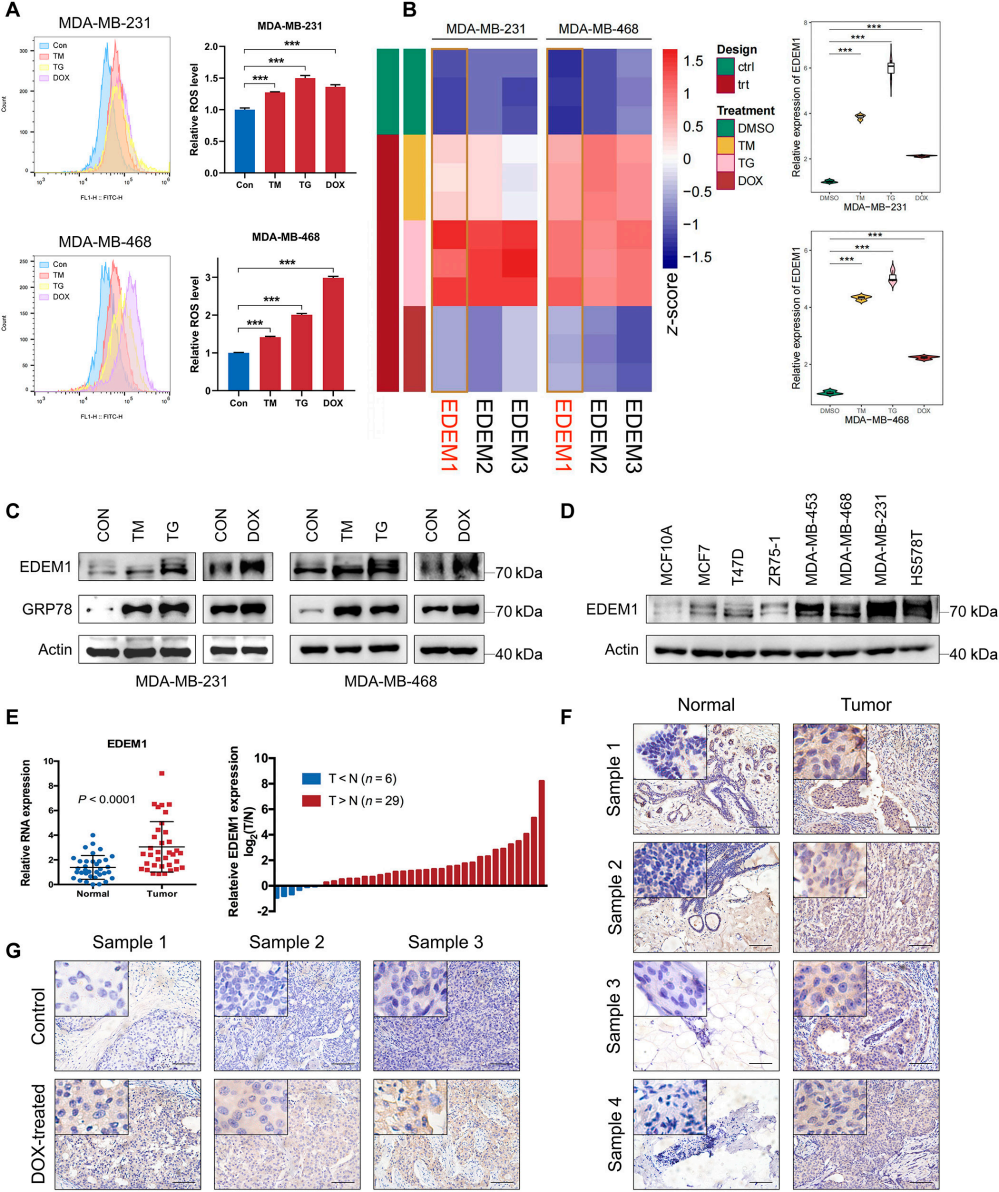
EDEM1 plays a crucial part during ER stress induced by doxorubicin and is highly expressed in breast cancer. (A) Flow cytometry analysis of ROS levels in MDA-MB-231 and MDA-MB-468 cells treated with TM (5 μg/ml), TG (1 μM), or DOX (1 μM) for 24 h. (B) Relative mRNA fold change of all EDEM family members in MDA-MB-231 and MDA-MB-468 cells treated with TM (5 μg/ml), TG (1 μM), or DOX (1 μM) for 24 h. (C) Western blot analysis of the levels of EDEM1 and GRP78 in MDA-MB-231 and MDA-MB-468 cells treated with TM (5 μg/ml), TG (1 μM), or DOX (1 μM) for 24 h. (D) Western blot analysis the levels of EDEM1 in human normal mammary epithelial cells (MCF-10A) and breast cancer cell lines (MCF-7, T47D, ZR75-1, MDA-MB-453, MDA-MB-468, MDA-MB-231, and HS578T). (E) Relative mRNA fold change of EDEM1 in normal breast tissue and breast cancer tissue of patients in Qilu Hospital. (F) IHC analysis of EDEM1 expression in breast cancer tissue and adjacent normal breast tissue of patients in Qilu Hospital. (G) IHC analysis of EDEM1 expression in DOX-treated breast cancer tissues and non-DOX-treated breast cancer tissues of patients in Qilu Hospital. Scale bar = 100 μm (*P < 0.05, **P < 0.01, ***P < 0.001).

During the accumulation of misfolded proteins in the ER lumen, the UPR response accelerates the reactive oxygen species (ROS) production in the ER, which is a prominent event during ER stress [32]. Therefore, we first examined the cellular ROS levels in MDA-MB-231 and MDA-MB-468 cell lines in normal conditions and tunicamycin (TM), thapsigargin (TG), and DOX-treated conditions. Results of flow cytometry analysis showed that ROS levels were substantially increased after TM, TG, and DOX treatment (Fig. 1A), suggesting the induced redox imbalance and ER stress by chemotherapy drugs. To explore the role of EDEM family members in the ER stress induced by DOX, we examined the expression levels of all EDEM genes (EDEM1 to EDEM3) in TNBC cells (MDA-MB-231/MDA-MB-468) following treatment with ER stress-inducing drugs, TM or TG, and DOX. Results of quantitative real-time PCR (qRT-PCR) showed that all EDEM genes were up-regulated (Fig. 1B). Given the critical role of EDEM1 in ERAD and its unique functional attributes, we focused on EDEM1 for further investigation. Western blotting verified that the protein level of EDEM1 was indeed increased upon treatment with TM, TG, or DOX in MDA-MB-231 and MDA-MB-468 cell lines. Concurrently, the expression of GRP78, one of the ER stress markers used as the positive control [33], was also substantially increased, suggesting the successful induction of ER stress (Fig. 1C).

To investigate the potential roles of EDEM1 in breast cancer, we examined its differential expression between normal cells and breast cancer cells and found that EDEM1 is highly expressed in most breast cancer cells compared with human normal mammary epithelial cells (MCF-10A). Besides, the expression of EDEM1 was higher in TNBC cell lines than in non-TNBC cell lines (Fig. 1D). We further detected the differential expression of EDEM1 between human breast cancer tissues and normal tissues in The Cancer Genome Atlas and Metabric databases, and up-regulated mRNA expression of EDEM1 was found in breast cancer tissues (Fig. S1A). Similar results were also obtained in tissues from our cohort: EDEM1 was increased in the majority of the breast cancer tissues (Fig. 1E). Uniformly, the immunohistochemistry (IHC) analysis was performed on tumor tissues and adjacent normal tissues, and proved that EDEM1 was up-regulated in breast cancer tissues in most patients (23/25) (Fig. 1F). Besides, EDEM1 expression was also elevated in breast cancer tissues treated with DOX during neoadjuvant chemotherapy (Fig. 1G), further strengthening the relationship between EDEM1 expression and DOX resistance. All the above results suggested that EDEM1 expression was induced to increase during ER stress and plays potentially important roles in breast cancer progression and chemoresistance.

### EDEM1 promotes progression and chemoresistance of TNBC cells

Considering the up-regulated EDEM1 expression in breast cancer, we wonder whether EDEM1 served as a tumor promoter in TNBC. MDA-MB-231 and MDA-MB-468, 2 widely used TNBC cell lines, were chosen to further validate the functional roles of EDEM1. We up-regulated EDEM1 expression with Flag-EDEM1 plasmid and verified the overexpression efficiency by qRT-PCR (Fig. S1B). The 3-(4,5-dimethylthiazol-2-yl)-2,5-diphenyltetrazolium bromide (MTT) assays indicated that EDEM1 overexpression enhanced the proliferation abilities of TNBC cells (Fig. S1C). The 5-ethynyl-2'-deoxyuridine (EdU) assays and flow cytometry analysis further determined that EDEM1 overexpression promoted cell cycle progression (Fig. S1D and E). Transwell and wound healing assays showed that EDEM1 overexpression markedly promoted the migration and invasion abilities of TNBC cells (Fig. S1F and G). Meanwhile, EDEM1 knockdown inhibited cell proliferation and metastasis abilities in TNBC cells (Fig. S2), indicating the oncogenic role of EDEM1.

**Fig. 2.**
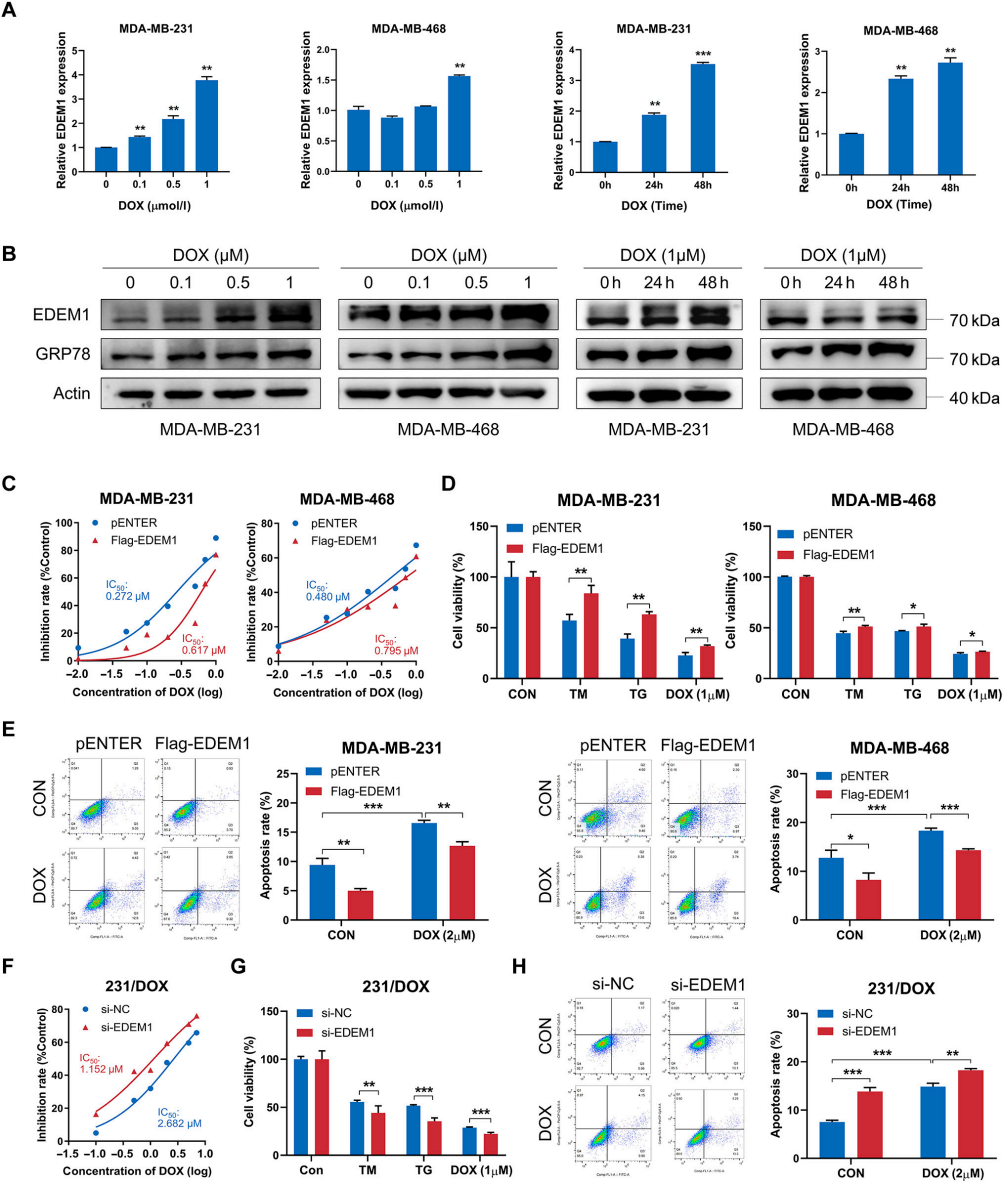
EDEM1 promotes doxorubicin resistance of TNBC cells. (A) Relative mRNA fold change of EDEM1 in MDA-MB-231 and MDA-MB-468 cells treated with a gradient concentration of DOX for 24 h or treated with DOX (1 μM) for gradient time. (B) Western blot analysis of EDEM1 and GRP78 protein levels in MDA-MB-231 and MDA-MB-468 cells treated with a gradient concentration of DOX for 24 h or treated with DOX (1 μM) for a gradient time. (C) Inhibitory curve of control and EDEM1-overexpressing MDA-MB-231 and MDA-MB-468 cells pretreated with different concentrations of DOX for 48 h. (D) Cell viability of control and EDEM1-expressing MDA-MB-231 and MDA-MB-468 cells pretreated with TM (5 μg/ml), TG (1 μM), or DOX (2 μM) for 24 h. (E) Flow cytometry analysis of apoptosis rates in control and EDEM1-expressing MDA-MB-231 and MDA-MB-468 cells pretreated with DOX (2 μM) for 24 h. (F) Inhibitory curve of control and EDEM1-knowdown 231/DOX cells pretreated with different concentrations of DOX for 48 h. (G) Cell viability of control and EDEM1-knowdown 231/DOX cells pretreated with TM (5 μg/ml), TG (1 μM), or DOX (1 μM) for 24 h. (H) Flow cytometry analysis of apoptosis rates in control and EDEM1-knowdown 231/DOX cells pretreated with DOX (2 μM) for 24 h (ns, no significance, *P < 0.05, **P < 0.01, ***P < 0.001).

In addition, we found that the expression level of EDEM1 was up-regulated in DOX-resistant cells (231/DOX) compared with parental MDA-MB-231 cells (Fig. S3A and B). Knockdown of EDEM1 could attenuate the proliferation and metastasis abilities of 231/DOX cells (Fig. S3C to G) and promote cell apoptosis (Fig. S3H). Besides, elevated mRNA and protein expression levels of EDEM1 were found after DOX treatment in a dose- and time-dependent manner (Fig. 2A and B), highlighting the potential role of EDEM1 in DOX resistance. Significantly, compared with control cells, the IC_50_ value of DOX was higher after EDEM1 overexpression in TNBC cells (Fig. 2C). Moreover, EDEM1 overexpression could recover the cell viability of TNBC cells after DOX treatment (Fig. 2D). Flow cytometry analysis also found that the apoptotic rates were decreased after EDEM1 overexpression in both control and DOX treatment conditions (Fig. 2E). Consistently, EDEM1 knockdown led to a decreased IC_50_ value of DOX (Fig. 2F), inhibited cell viability (Fig. 2G), and increased apoptosis (Fig. 2H) in 231/DOX cells after DOX treatment. Together, these results provided convincing evidence that EDEM1 enhances DOX resistance in TNBC cells.

### EDEM1 confers DOX resistance by facilitating ERAD and reducing ER stress-induced ROS production in TNBC cells

**Fig. 3.**
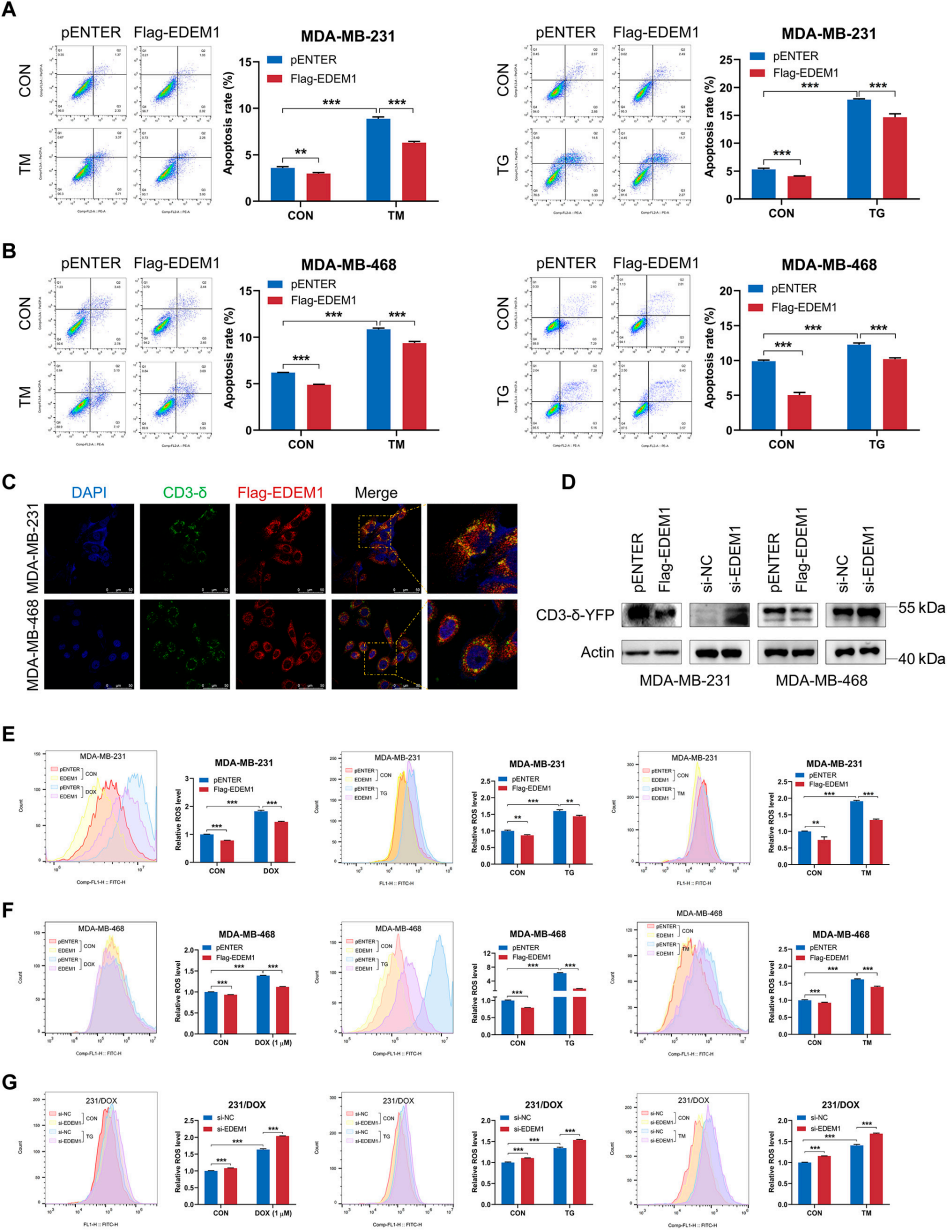
EDEM1 promotes TNBC chemoresistance via facilitating ERAD and reducing ER stress-induced ROS levels. (A) Flow cytometry analysis of apoptosis rates in control and EDEM1-overexpressing MDA-MB-231 cells pretreated with TM (5 μg/ml) and TG (1 μM) for 24 h. (B) Flow cytometry analysis of apoptosis rates in control and EDEM1-overexpressing MDA-MB-468 cells pretreated with TM (5 μg/ml) and TG (1 μM) for 24 h. (C) Colocalization of CD3-δ and EDEM1 in MDA-MB-231 and MDA-MB-468 cells. Scale bar = 50 μm. (D) Western blot analysis of the levels of CD3-δ-YFP in MDA-MB-231 and MDA-MB-468 cells treated with EDEM1 overexpression or knockdown. (E) Flow cytometry analysis of ROS levels in control and EDEM1-overexpressing MDA-MB-231 cells treated with DOX (1 μM), TM (5 μg/ml), or TG (1 μM) for 24 h. (F) Flow cytometry analysis of ROS levels in control and EDEM1-overexpressing MDA-MB-468 cells treated with DOX (1 μM), TM (5 μg/ml), or TG (1 μM) for 24 h. (G) Flow cytometry analysis of ROS levels in control and EDEM1-knockdown 231/DOX cells treated with DOX (1 μM), TM (5 μg/ml), or TG (1 μM) for 24 h (ns, no significance, *P < 0.05, **P < 0.01, ***P < 0.001).

Meanwhile, we found that DOX treatment led to increased expression of GRP78 in a dose- and time-dependent manner in TNBC cells (Fig. 2B), which is similar to the expression change of EDEM1, further revealing the induction of ER stress after DOX treatment and the involvement of EDEM1 during DOX-induced ER stress. As expected, our results validated that EDEM1 overexpression led to decreased TM- or TG-induced growth inhibition and cell apoptosis (Figs. 2D and 3A and B), while EDEM1 knockdown caused opposite results (Fig. 2G and Fig. S4A). Previous studies reported that cell apoptosis would be triggered if ER stability is not restored after certain stimuli. Therefore, the inhibited apoptotic rates after EDEM1 overexpression prompt us to explore the underlying quality-control machinery that EDEM1 activates to relieve ER stress. We first examined the effect of EDEM1 on the UPR pathway. The mRNA expression levels of 2 UPR downstream genes, sXBP1 and ATF6, were slightly up-regulated upon EDEM1 alteration, and no obvious changes were observed in ATF4 and pro-apoptotoc C/EBP homologous protein (CHOP) (Fig. S4B), indicating that EDEM1 could sightly activate IRE1 and ATF6 pathways. We further investigated the role of EDEM1 in autophagy, which could be induced by ER stress [15]. Surprisingly, monodansulfonylpentanediamine (MDC) assay and immunofluorescence (IF) assay for LC3B showed that, in normal stress or starvation-induced stress conditions, EDEM1 overexpression could reduce the level of autophagy, while EDEM1 knockdown caused opposite results (Fig. S5). Combining with the previous results, we speculated that EDEM1 might reduce the ER stress of cells through other mechanisms, thus leading to the reduction of autophagy. Given the role of EDEM1 in ERAD, a crucial quality control mechanism for maintaining ER homeostasis, we further determined whether EDEM1 contributed to the clearance of misfolded proteins through ERAD beforehand to prevent ER stress. CD3-δ-YFP, an ER marker and classical ERAD substrate [34], is widely used as a common reporter to assess ERAD efficiency [35]. IF assay indicated that EDEM1 could colocalize with CD3-δ-YFP (Fig. 3C), providing a prerequisite for the regulation of EDEM1 on the expression of CD3-δ-YFP. Indeed, overexpression of EDEM1 decreased CD3-δ-YFP protein level, while EDEM1 knockdown led to increased protein abundance of CD3-δ-YFP (Fig. 3D), indicating enhanced ERAD activity in the presence of EDEM1.

Interestingly, we also found that the cellular ROS levels, a prominent event during ER stress [36], were increased after TM, TG, and DOX treatment (Fig. 1A). Given the association between EDEM1 and ER stress, we wonder whether EDEM1 could modulate cellular ROS production in tumor cells. Results of flow cytometry analysis showed that overexpression of EDEM1 slightly attenuated ROS accumulation in TNBC cells in the control condition, and markedly attenuated ROS accumulation in stressed conditions induced by TM, TG, and DOX (Fig. 3E and F). Meanwhile, depletion of EDEM1 markedly increased ROS production in the 231/DOX cells under both control conditions and TM-, TG-, and DOX-treated conditions (Fig. 3G). These results indicated that EDEM1 elevation confers increased ability in governing redox balance to maintain ER homeostasis in both nonstressed and stressed conditions, leading to enhanced drug resistance of TNBC cells. Taken together, these data here suggest that EDEM1 maintains ER homeostasis via accelerating the ERAD process and governing redox balance.

### EDEM1 positively regulates the antioxidant capacity of TNBC cells via stabilizing Nrf2 and promoting its nuclear translocation

**Fig. 4.**
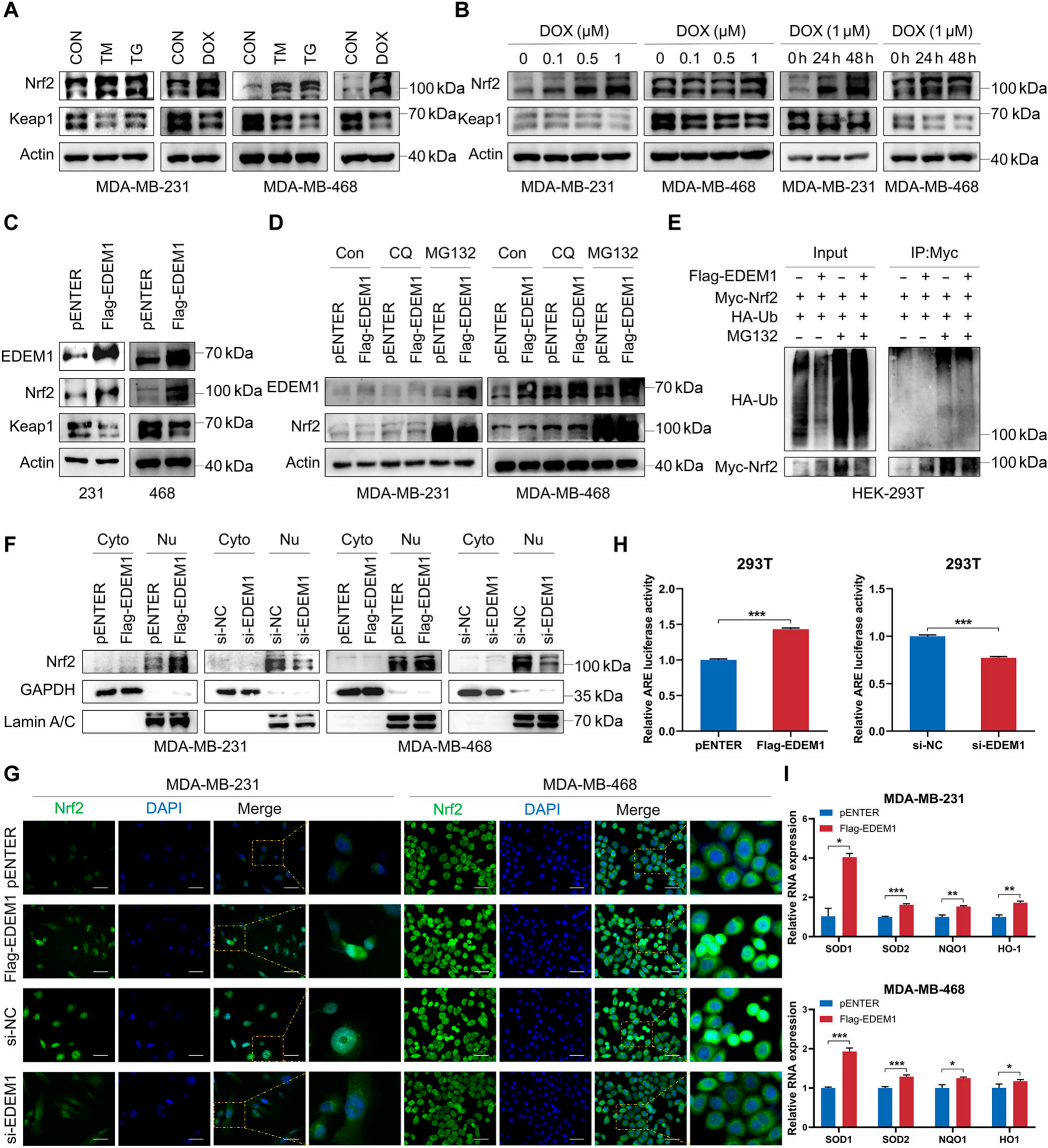
EDEM1 positively regulates the antioxidant capacity of TNBC cells via stabilizing Nrf2 and promoting its nuclear import. (A) Western blot analysis of Nrf2 and Keap1 levels in MDA-MB-231 and MDA-MB-468 cells treated with TM (5 μg/ml), TG (1 μM), or DOX (1 μM) for 24 h. (B) Western blot analysis of Nrf2 and Keap1 levels in MDA-MB-231 and MDA-MB-468 cells treated with a gradient concentration of DOX for 24 h or treated with DOX (1 μM) for gradient time. (C) Western blot analysis of EDEM1, Nrf2, and Keap1 levels in control and EDEM1-overexpressing MDA-MB-231 and MDA-MB-468 cells. (D) Western blot analysis of EDEM1 and Nrf2 levels in control and EDEM1-overexpressing MDA-MB-231 and MDA-MB-468 cells treated with CQ (20 μM) and MG132 (10 μM) for 12 h. (E) HEK-293T cells transfected with the plasmids as indicated. Immunoblot analyses of cell lysates and Myc-IP with indicated antibodies were conducted. (F) The level of Nrf2 in the cytoplasmic and nuclear fractions in control and EDEM1-overexpressing or EDEM1-knockdown MDA-MB-231 and MDA-MB-468 cells. (G) Localization of Nrf2 (green) in control and EDEM1-overexpressing or EDEM1-knockdown MDA-MB-231 and MDA-MB-468 cells. The nuclei are labeled by DAPI (blue). Scale bar = 50 μm. (H) Dual-luciferase reporter assay of ARE transcriptional activity in control and EDEM1-overexpressing or EDEM1-knockdown HEK-293T cells. (I) Relative mRNA fold change of SOD1, SOD2, NQO1, and HO-1 in control and EDEM1-overexpressing MDA-MB-231 and MDA-MB-468 cells (*P < 0.05, **P < 0.01, ***P < 0.001).

The Keap1/Nrf2 pathway is a crucial and highly conserved defense system required to maintain or restore the intracellular homeostasis under oxidative, electrophilic, and other types of stress conditions, which is associated with metabolic reprogramming, increased antioxidant capacity, chemoresistance, and poor clinical outcome [37]. To explore whether the Keap1/Nrf2 pathway plays a vital role in DOX-induced ER stress, we analyzed the Keap1, Nrf2 expression in TNBC cells after TM, TG, and DOX treatment. Decreased Keap1 and elevated Nrf2 expression were found in TNBC cells induced by all these treatments and were found in a dose- and time-dependent manner (Fig. 4A and B), indicating the crucial role of the Keap1/Nrf2 pathway in DOX resistance and during DOX-induced ER stress of TNBC. Then, we speculated whether EDEM1 plays its role in chemoresistance via promoting the Keap1/Nrf2 pathway. Results demonstrated that EDEM1 overexpression led to decreased expression of Keap1 and increased expression of Nrf2 (Fig. 4C). We further examined the role of EDEM1 in regulating the protein stability of Nrf2. Chloroquine (CQ, a lysosome inhibitor) and MG132 (a proteasome inhibitor) were used to treat TNBC cells to investigate the possibility of its lysosomal and proteasomal degradation. The results showed that MG132 led to up-regulated Nrf2 compared to the other 2 groups (Fig. 4D). The above results indicated that EDEM1 activated the Keap1/Nrf2 pathway and up-regulated Nrf2 through inhibiting its ubiquitin-mediated proteasome degradation. The co-IP results showed that EDEM1 overexpression decreased the ubiquitination levels of Nrf2 (Fig. 4E). Previous studies have proved that the intranuclear translocation of Nrf2 is related to its activation of downstream gene transcription that encodes antioxidant enzymes, which protect cells from oxidative damage [38]. In our study, the levels of the nuclear fraction of Nrf2 protein examined by both Western blot and IF were found notably increased after EDEM1 overexpression and decreased after EDEM1 knockdown (Fig. 4F and G). Since Nrf2 regulates antioxidant enzymes such as HO-1 and others via moving to the nucleus and binding to the ARE sequence [39], we conducted an ARE-Luc reporter assay and proved that EDEM1 activated ARE signaling through Nrf2 (Fig. 4H). Consistently, the mRNA expressions of Nrf2 downstream target genes including SOD1, SOD2, NQO1, and HO-1 were also activated upon EDEM1 overexpression (Fig. 4I). These results demonstrated that EDEM1 positively regulated the antioxidant capacity of tumor cells through activating the Keap1/Nrf2/ARE pathway. This effective activation was realized by preventing Nrf2 from ubiquitination and degradation, and promoting nuclear import and ARE recognition of Nrf2.

### EDEM1 prevents Nrf2 from ubiquitin-mediated proteasome degradation by competitive binding Keap1

**Fig. 5.**
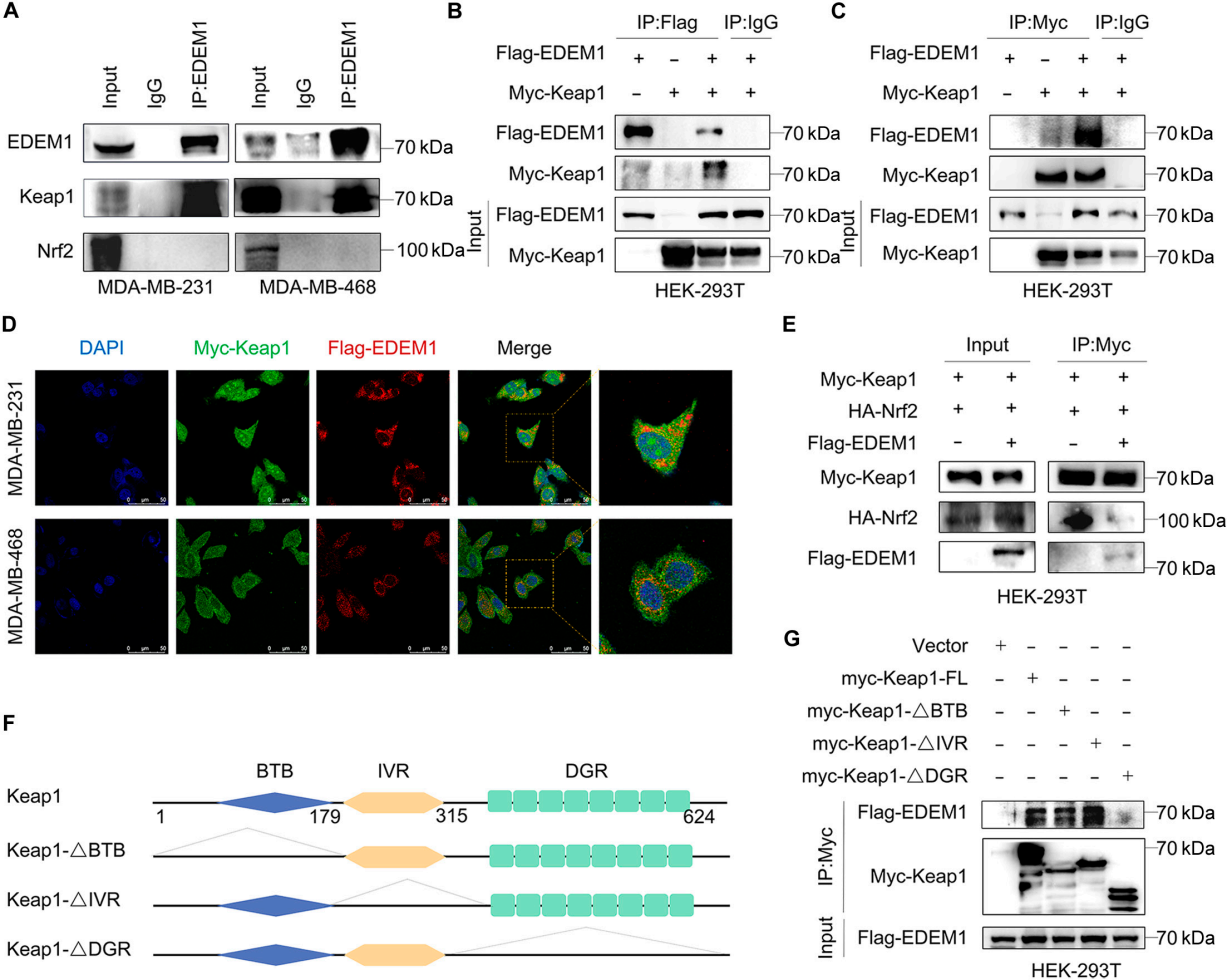
EDEM1 prevents Nrf2 from ubiquitination by competitively binding Keap1. (A) Co-immunoprecipitation (co-IP) assay analyzed the interaction of endogenous EDEM1 and Keap1 in MDA-MB-231 and MDA-MB-468 cells. (B) HEK-293T cells were transfected with the indicated plasmids. Immunoblot analyses of the Flag-IP and cell lysates were conducted. (C) HEK-293T cells were transfected with the indicated plasmids. Immunoblot analyses of the Myc-IP and cell lysates were conducted. (D) Colocalization of Keap1 (green) and EDEM1 (red) in MDA-MB-231 and MDA-MB-468 cells. Scale bar = 50 μm. The nuclei are labeled by DAPI (blue). (E) HEK-293T cells were transfected with the indicated plasmids. Immunoblot analysis of the Myc-IP and cell lysates was conducted. (F) The BTB, IVR, and DGR domains of Keap1 were indicated. (G) HEK-293T cells were transfected with the indicated plasmids. Immunoblot analyses of the Myc-IP and cell lysates were conducted.

Since the ubiquitination of Nrf2 is regulated by EDEM1, we speculated that EDEM1 might affect the interaction between Nrf2 and Keap1, the major E3 ligase of Nrf2 [40]. Thus, we first performed an endogenous immunoprecipitation (IP) assay to examine the combination between EDEM1 and Keap1 or Nrf2 within TNBC cell lysates. The results showed that EDEM1 bound to endogenous Keap1 instead of Nrf2 (Fig. 5A). Then, the co-immunoprecipitation (co-IP) assay between Flag-EDEM1 and Myc-Keap1 validated their interactions as well (Fig. 5B and C). The IF assay also revealed the same result that EDEM1 colocalized with Keap1 (Fig. 5D). Importantly, the physical interaction between Nrf2 and Keap1 was obviously reduced when EDEM1 was coexpressed in human embryonic kidney (HEK)-293T cells (Fig. 5E), suggesting that EDEM1 competitively bound with Keap1 to prevent Nrf2 from protein degradation through the ubiquitin-mediated proteasome signaling pathway. Next, we generated a series of truncated mutants of Keap1 to dissect the functional domains of the EDEM1–Keap1 interaction and found that the DGR domain of Keap1 was crucial for its interaction with EDEM1 (Fig. 5F and G). Taken above, our data revealed that EDEM1 prevented Nrf2 from ubiquitination and degradation by competitively binding the DGR domain of Keap1.

### Upstream miR-32-5p down-regulates the expression and function of EDEM1 in TNBC

**Fig. 6.**
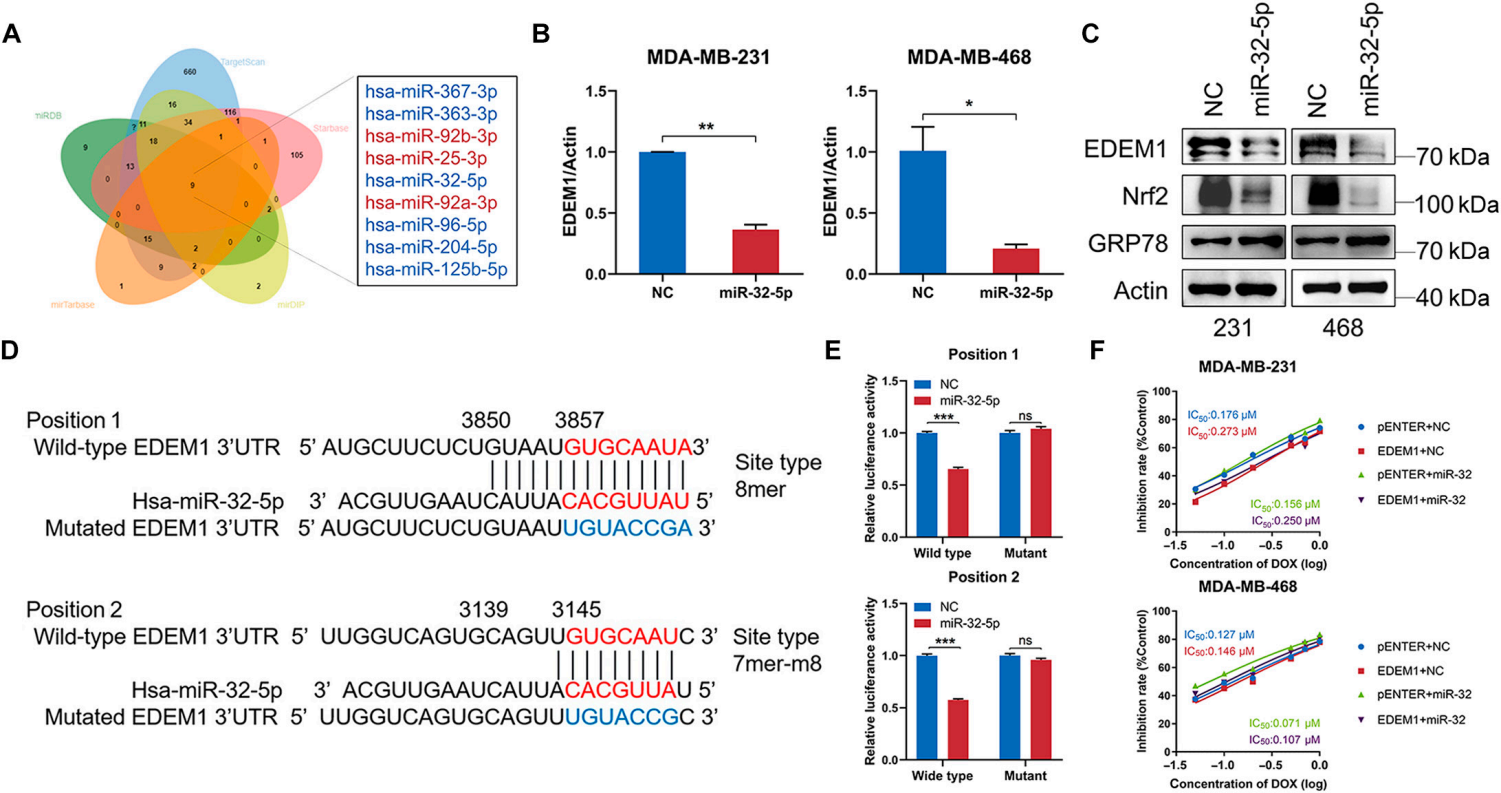
EDEM1 mediates TNBC progression and chemoresistance targeted by miR-32-5p. (A) Nine potential target miRNAs of EDEM1 were predicted by TargetScan, Starbase, mirDIP, mirTarbase, and miRDB. (B) Relative mRNA fold change of EDEM1 in MDA-MB-231 and MDA-MB-468 cells transfected with NC or miR-32-5p mimics. (C) Western blot analysis of EDEM1 levels and Nrf2 levels in control and miR-32-5p overexpression MDA-MB-231 and MDA-MB-468 cells. (D) Schematic representation of 2 binding sites’ wild-type (wt) and mutant (mut) luciferase reporter vectors. (E) Luciferase reporter assay in HEK-293T cells cotransfected with miRNA mimics, Position 1-Wild type and Mutant, and Position 2-Wild type and Mutant plasmids. (F) Inhibitory curve of MDA-MB-231 and MDA-MB-468 cells transfected with indicated plasmids and mimics and pretreated with different concentrations of DOX for 48 h (ns, no significance, *P < 0.05, **P < 0.01, ***P < 0.001).

We further sought molecules that could target EDEM1 to overcome resistance. Given that microRNAs (miRNAs) are well-documented for their ability to inhibit gene expression through posttranscriptional regulation of protein accumulation [41] and their established role in tumor development and progression [42], we focused on investigating whether EDEM1 is regulated by miRNAs. Utilizing several predictive tools (Fig. 6A), we explored potential miRNAs that may bind to and regulate EDEM1 expression and function. Through consulting literature, public database analysis in KM plotter (https://kmplot.com/analysis/), and qPCR verification, it was found that high miR-32-5p expression was correlated with better prognosis of breast cancer patients and decreased in most breast cancer cells compared with normal cell (MCF-10A) (Fig. S6A and B). Moreover, the expression of miR-32-5p was negatively correlated with EDEM1 expression in cancer cells, consistent with the predicted results by the StarBase database (Fig. S6C and D). Therefore, miR-32-5p was selected as the upstream regulator of EDEM1 for further investigation. We initially confirmed that miR-32-5p overexpression down-regulated EDEM1 at the RNA level and simultaneously reduced the protein levels of both EDEM1 and Nrf2 (Fig. 6B and C). Using the publicly available bioinformatics tool TargetScan, we found that 3′UTR of EDEM1 contains 2 putative miR-32-5p binding sites (Fig. 6D). To further confirm that EDEM1 was a target of miR-32-5p, luciferase assays were performed to verify the interaction between miR-32-5p and EDEM1. EDEM1 3′UTR fragment containing the wild-type and mutant binding sites with miR-32-5p was cloned into the luciferase reporter plasmid pmirGLO, and cotransfected with miR-32-5p mimic or miR-NC mimic into 293T cells. The results showed that overexpression of miR-32-5p prominently diminished the luciferase activity of the vector including the wild-type binding sites but not the mutant binding sites (Fig. 6E). These findings suggested that miR-32-5p could bind 3′UTR of EDEM1 to down-regulate its expression.

We further explore the functional roles of miR-32-5p in TNBC cells. We up-regulated miR-32-5p expression and verified the overexpression efficiency by qRT-PCR (Fig. S7A). The MTT assay indicated that miR-32-5p overexpression suppressed the proliferation abilities of TNBC cells (Fig. S7B). EdU assays also determined that miR-32-5p decreased DNA synthesis activities (Fig. S7C). Transwell and wound healing assays showed that miR-32-5p notably attenuated the migration and invasion abilities of TNBC cells (Fig. S7D and E). Flow cytometry analysis also showed that miR-32-5p overexpression promoted apoptosis of TNBC cells in vitro (Fig. S7F). Meanwhile, we also proved that miR-32-5p overexpression decreased the IC_50_ value of TNBC cells (Fig. S7G). These results indicated that miR-32-5p suppressed TNBC progression and chemoresistance. To analyze whether miR-32-5p modulates TNBC progression through targeting EDEM1, rescue experiments were performed, which further proved that miR-32-5p suppressed the progression and chemoresistance of TNBC through down-regulating EDEM1 (Fig. 6F and Fig. S8). These findings position miR-32-5p as a potential molecule that could inhibit EDEM1 to overcome chemoresistance.

### EDEM1 promotes TNBC progression and chemoresistance in vivo

**Fig. 7.**
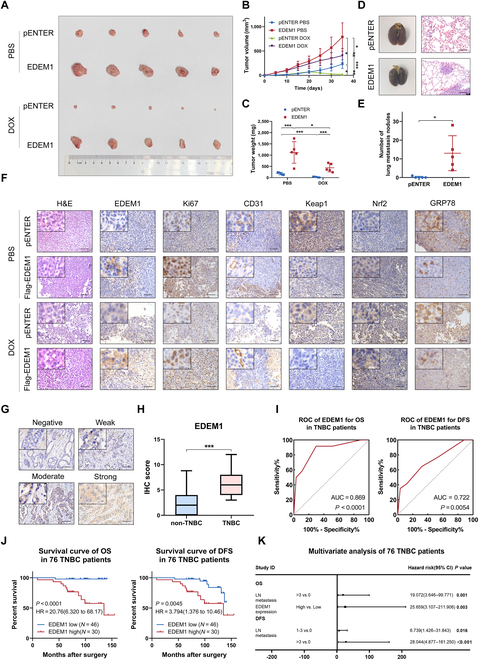
EDEM1 promotes TNBC progression and chemoresistance in vivo and is correlated with poor prognosis of TNBC. (A) The image of tumors excised from all nude mice at day 35. (B and C) Tumor mass (B) and tumor volume (C) are measured in MDA-MB-231 xenograft. (D) The representative image of lung metastatic nodules and H&E staining of lung. Scale bar = 100 μm. (E) Statistical chart of the number of lung metastatic nodules. (F) The representative images for H&E staining and immunohistochemical staining of EDEM1, Ki67, CD31, Keap1, Nrf2, and GRP78 in tumor tissues. Scale bar = 100 μm. (G) The representative images were determined by IHC scoring. Scale bar = 100 μm. (H) IHC scores differences of EDEM1 between non-TNBC patients and TNBC patients. (I) ROC analysis of OS and DFS between high EDEM1 expression and low EDEM1 expression TNBC patients. (J) Log-rank test of OS and DFS between high EDEM1 expression and low EDEM1 expression TNBC patients. (K) Forest plot of independent risk factors in multivariate Cox analysis for OS and DFS of 76 TNBC patients (*P < 0.05, **P < 0.01, ***P < 0.001).

To examine the role of EDEM1 in vivo, we explanted control and stable EDEM1-overexpressing MDA-MB-231 cells in nude mice to build a mouse xenograft model. When the tumor volume reached 50 mm^3^, phosphate buffered saline (PBS) or DOX was injected intravenously. Compared to the control, the EDEM1 overexpression group displayed markedly larger tumors in nude mice in both the PBS subgroup and DOX injection subgroup. Besides, the tumors in DOX-injected groups were overall smaller and lighter than the PBS-injected group, confirming that DOX suppressed the growth of breast cancer in vivo. Furthermore, though DOX suppressed the growth of breast cancer cells, these suppressive effects were more substantial in the control group rather than the EDEM1 overexpression group, which indicated that EDEM1 could attenuate the anticancer effect of DOX (Fig. 7A to C). In order to validate the pro-metastasis effect of EDEM1 in vivo, we injected the control or EDEM1 stable-overexpression MDA-MB-231 cells into nude mice via the tail vein to construct a pulmonary metastasis model. The results showed that the number of lung metastatic nodules in the EDEM1 overexpression group was more than that of the control group. Lung metastases were verified by histological staining (Fig. 7D and E). The morphology of xenograft tumors was also evaluated by H&E staining (Fig. 7F). The IHC analyses of Ki67 and CD31 demonstrated that EDEM1 overexpression promoted tumor proliferation and angiogenesis. The terminal deoxynucleotidyl transferase-mediated dUTP nick end labeling (TUNEL) assay validated that EDEM1 overexpression inhibited tumor apoptosis both in normal conditions and in DOX-induced conditions (Fig. S9A). Moreover, in contrast to the control tumor xenografts, a reduction in Keap1 expression and up-regulation of Nrf2 expression were observed in the tumor xenografts derived from MDA-MB-231 cells with EDEM1 overexpression both in normal conditions and in DOX-induced conditions, suggesting that the EDEM1-induced Keap1–Nrf2 pathway plays a vital role in TNBC progression and chemoresistance (Fig. 7F). Also, IHC staining of GRP78 confirmed that DOX enhanced ER stress in tumor tissue, while the overexpression of EDEM1 attenuated the ER stress level induced by DOX (Fig. 7F). Rescue experiments in vitro and in vivo were performed, which further proved that EDEM1 promoted the proliferation and chemoresistance of TNBC through targeting Nrf2 (Fig. S9B to E). The above results demonstrate that EDEM1 attenuates ER stress in cancer cells to promote TNBC progression and chemoresistance in vivo through Keap1/Nrf2 signaling.

### Up-regulation of EDEM1 correlates with poor clinical breast cancer prognosis

To reveal the clinical prognostic prediction value of EDEM1 in breast cancer, we collected tumor tissues from 131 breast cancer patients. We gathered the clinical features and prognosis information of these 131 breast cancer patients and performed IHC staining to evaluate EDEM1 expression in these breast cancer tissues. Firstly, Kaplan–Meier analysis showed that breast cancer patients with high EDEM1 expression had poor overall survival (OS) and disease-free survival (DFS) (Fig. 7G and Fig. S10A and B). Then, the chi-square test was used to evaluate the correlations between EDEM1 expression and clinicopathologic features of breast cancer patients, including age, tumor stage, LN metastasis, distant metastasis, histologic grade, ER (estrogen receptor) status, PR (progesterone receptor) status, HER-2 status, and Ki67 expression. The results indicated that high expression of EDEM1 was prominently associated with ER status and PR status in breast cancer (*P* < 0.001), indicating a potential role of EDEM1 in breast cancer progression, especially TNBC progression (Table S1). Univariate and multivariate Cox regression analyses were then performed to screen the potential prognostic factors in breast cancer. We found that >3 LN metastases and EDEM1 expression were independent prognostic risk factors for the OS of breast cancer (Fig. S10C and Table S2). Meanwhile, >3 LN metastases, distant metastasis, and EDEM1 expression were proved to be independent prognostic indicators for DFS of breast cancer (Fig. S10C and Table S3). These results indicated that EDEM1 was an unfavorable prognostic biomarker in breast cancer.

Considering that EDEM1 expression was closely associated with ER and PR expression, we compared the IHC score between non-TNBC patients and TNBC patients. The results showed that the IHC score of EDEM1 expression in TNBC patients was notably higher than that in non-TNBC patients (Fig. 7H). Kaplan–Meier analysis showed that high EDEM1 expression predicted poor OS and DFS of TNBC patients (Fig. 7I and J). Then, a chi-square test was used to evaluate the correlations between EDEM1 expression and clinicopathologic features of breast cancer patients, and no noticeable difference was found between the 2 groups (Table S4). Furthermore, univariate and multivariate Cox regression analyses were performed to screen the prognostic factors in TNBC. We found that >3 LN metastasis and EDEM1 expression were independent prognostic risk factors for the OS of TNBC (Fig. 7K and Table S5). Meanwhile, 1 to 3 LN metastases and >3 LN metastases were proved to be independent prognostic indicators for DFS of TNBC (Fig. 7K and Table S6). Taken together, the above findings indicated that elevated levels of EDEM1 contribute to tumor progression and serve as an important indicator for poor prognosis in breast cancer patients, thus providing a potential therapeutic target for cancer prevention.

## Discussion

TNBC is a distinct molecular subtype of breast cancer, which is highly invasive and heterogeneous [43]. Due to the lack of estrogen receptor, progesterone receptor, and HER2 expression/amplification, TNBC patients cannot benefit from endocrine therapy or anti-HER2 therapy, which limits therapeutic options and exhibits a more aggressive clinical behavior. However, the underlying mechanisms driving this malignancy are complex and multifaceted, involving various key regulatory molecules and signaling pathways that operate at multiple biological levels. For instance, POP1 has been shown to promote TNBC cell proliferation by facilitating m6A-dependent degradation of CDKN1A mRNA [44]. In addition, posttranslational modifications [45], such as phosphorylation and ubiquitination, further influence the activation or stabilization of oncogenic proteins, thereby modulating tumor aggressiveness and therapeutic resistance. Therefore, identifying novel key molecules involved in TNBC progression and developing strategies to target them represent promising avenues for therapeutic intervention. Although advances in targeted therapy and immunotherapy have shown some promise in mitigating the aggressiveness of TNBC, only a limited subset of patients benefit from these therapeutics. Therefore, chemotherapy still represents a potentially curative mainstay of systemic treatment for TNBC [46]. However, the development of chemoresistance remains a major clinical challenge. Despite extensive research, reliable biomarkers to predict treatment response are still lacking, leaving many patients at risk of ineffective or unnecessary therapy [47].

**Fig. 8.**
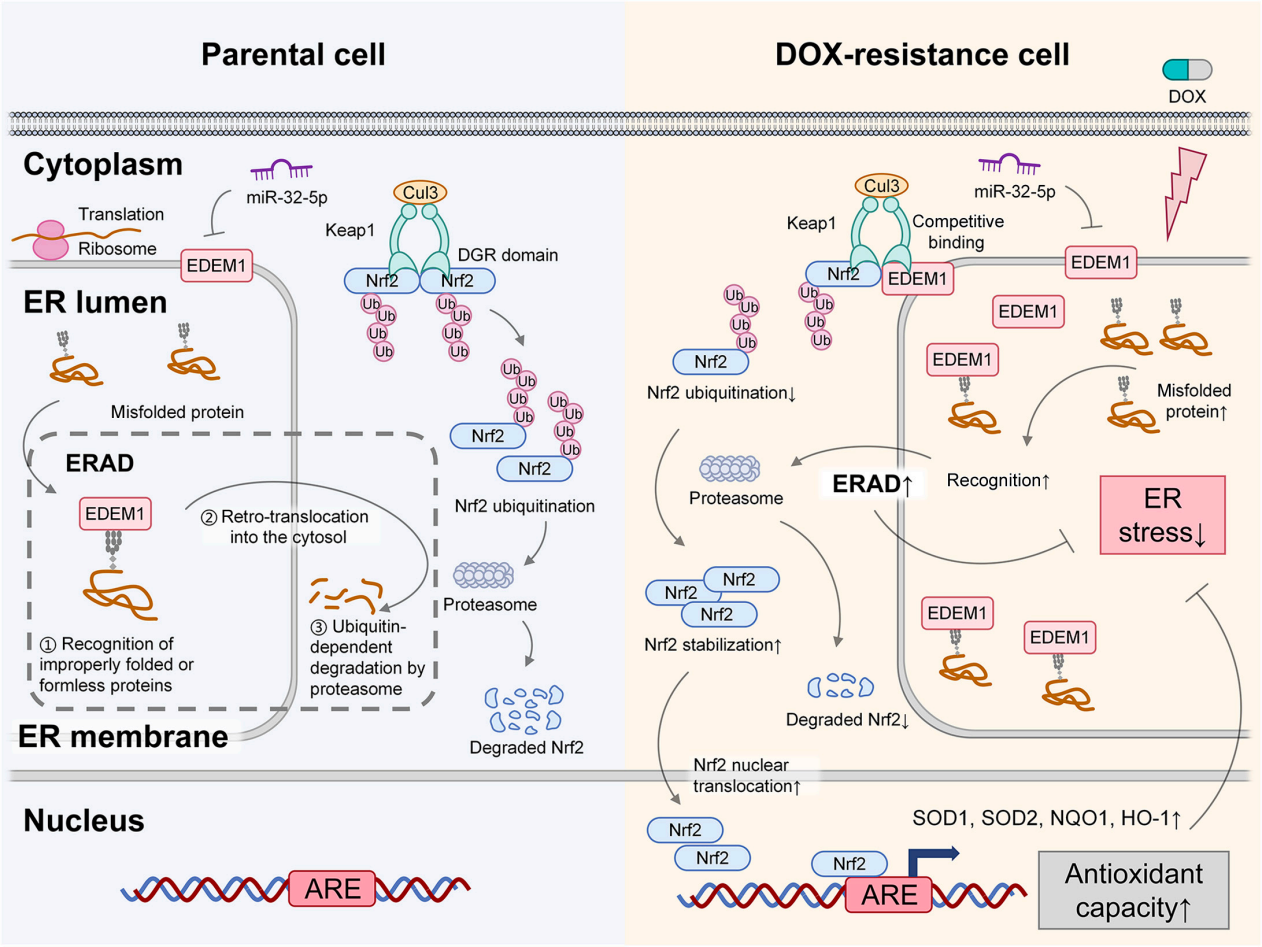
The mechanistic schematic models of the role of EDEM1 in doxorubicin resistance in TNBC cells.

Multiple mechanisms have been implicated in the acquisition of drug resistance, including reduced drug uptake, alterations in drug targets, activation of detoxification pathways, enhanced DNA repair capacity, enrichment of cancer stem cell (CSC), and insensitivity to drug-induced apoptosis [48]. These processes are often linked to molecular changes such as up-regulation of ATP-binding cassette transporters, dysregulation of signaling molecules such as tyrosine kinase receptors (e.g., EGFR and IGF-1R), a disintegrin and metalloproteinase 10 (ADAM10), noncoding RNAs, DNA methylation, and phosphoproteomic alterations [49]. For example, the mammalian target of rapamycin (mTOR) pathway interacts with multiple critical signaling cascades, such as PI3K/Akt, Notch, IGF-1R, AMPK, and TGF-β, as well as regulatory proteins and noncoding RNAs, forming a complex network that sustains CSC properties and contributes to therapeutic resistance [50]. Recent studies have also highlighted the role of ER stress in the development of chemoresistance. Chemotherapy can induce ER stress, and the subsequent activation of UPR signaling pathways has been shown to promote survival mechanisms in various tumor types [13,51], indicating that targeting ER stress-related signaling may offer a novel strategy to overcome chemoresistance. In this study, we explored the mechanism of chemoresistance from the perspective of chemotherapy-induced ER stress, screened out EDEM1 as a crucial regulator of DOX-induced ER stress, verified its role in promoting breast cancer progression and DOX resistance, and proved its prognostic value in breast cancer (Fig. 8).

The ER is a complex dynamic organelle that monitors a variety of cellular pathways, including protein synthesis, protein quality control, and lipid synthesis [52]. The fidelity of protein folding and maturation is critical for cell survival. Under normal conditions, unfolded or misfolded proteins are typically identified, selected, and localized to the ERAD complex. Certain physiological and pathological conditions can cause a high demand for secreted proteins or an imbalance between protein-folding demand and folding capabilities [53,54]. Then, the accumulation of unfolded or misfolded proteins activates the UPR to restore ER homeostasis or induces cell death under persistent ER stress [55], which is highly linked to many diseases including cancers [56]. Under different tumor-related conditions, diverse oncogenic, transcriptional, and metabolic abnormalities cooperate to generate hostile microenvironments that disrupt ER homeostasis, such as hypoxia, nutrient deprivation, ROS accumulation, and low pH [57]. However, tumor development is unaffected, indicating that tumor cells have complex mechanisms to acquire strong resistance to ER stress, which has not been fully elucidated. In addition, more and more researchers have found that ER stress induced by tumor cells plays an important regulatory role in tumor cell proliferation, apoptosis, and drug resistance [58]. Chemotherapy drugs can partially induce the ER stress process, and cells resistant to chemotherapy drugs also show resistance to ER stress [59], suggesting that resistance to ER stress of tumor cells may be one of the important mechanisms of chemoresistance. In our study, we first detected ER stress levels in TNBC cells after ER inducers (TM and TG) and DOX treatment, and found that the ER stress levels increased after all of the above treatments. Then, we wanted to seek ER stress-related molecules that play a key role in the DOX resistance of TNBC. Several studies proved that molecules related to ER stress can mediate chemoresistance in many tumors. For example, Heat shock factor 1 (HSF1) down-regulated mediated ER UPR can promote cisplatin resistance in lung cancer cells [60]. Melatonin reverses DOX-induced ER stress in human hepatocellular carcinoma cells by up-regulating CHOP and down-regulating survivin, thereby ameliorating DOX-induced cytotoxicity [61]. These findings suggest that it is important to screen out ER stress-related molecules or signaling pathways for further understanding of chemoresistance. We focused our research on the EDEM family, which plays an important role in the ERAD process. Three members of the EDEM family, EDEM1, EDEM2, and EDEM3, are vital in the ERQC process, which can accelerate the processing and degradation of misfolded proteins and promote the ERAD process [25]. However, the role of EDEM family members in TNBC chemoresistance has not been reported previously. In this study, after TNBC cells were treated with an ER stress inducer (TM and TG) and the chemotherapy drug DOX, EDEM1 had the highest up-regulation degree after various drug treatments. It was also found that the expression of EDEM1 was markedly up-regulated in both breast cancer cells and tissues. Therefore, we selected EDEM1 for further exploration.

Since the function of EDEM1 in breast cancer progression and chemoresistance has not been fully studied, we first carried out functional experiments in vitro and found that EDEM1 can promote the proliferation, metastasis, and DOX resistance of TNBC cells. To prove that EDEM1 is related to ER stress induced by DOX, we treated TNBC cells with DOX in gradient concentration and time and found that DOX can increase ER stress levels, which was consistent with previous studies [59]. At the same time, the expression of EDEM1 was gradually up-regulated. The up-regulation of EDEM1 enhanced tumor resistance to ER stress and DOX in TNBC cells, while the knockdown of EDEM1 exerted converse effects in DOX-resistant 231/DOX cells. Meanwhile, in vivo experiments using nude mice to construct subcutaneous xenograft and lung metastasis models also showed the same role of EDEM1 in TNBC progression, chemoresistance, and DOX-induced ER stress.

ER stress has been reported to induce not only UPR, ERAD, and autophagy [62] but also apoptosis when ER stability cannot be restored [15]. We then decided to validate the relationship between EDEM1 and these processes. Our study proved that EDEM1 can alleviate the autophagy of tumor cells and weaken the apoptosis process. Moreover, EDEM1 slightly activated the UPR pathway, accelerated ERAD, and thus promoted the degradation of misfolded proteins. Meanwhile, studies have shown that drugs can cause oxidative stress, and ER stress is closely related to oxidative stress. Overproduction of ROS promotes the accumulation of misfolded proteins and thus induces ER stress. At the same time, ER stress enhances the production of ROS, forming a pathological cycle [63]. Therefore, ROS level can be used as a marker to detect ER stress levels. We found that EDEM1 can slightly reduce ROS and substantially reduce ROS triggered by ER stress inducers and DOX. The above results confirmed the vital roles of EDEM1 in relieving DOX-induced ER stress, which was achieved by enhancing ERAD and alleviating oxidative stress.

We further explored the downstream mechanism by which EDEM1 exerts its function. The Keap1/Nrf2/ARE signaling pathway plays a vital role in protecting cells against endogenous and exogenous stresses, such as oxidative stress and xenobiotics, which can increase the antioxidant capacity of tumor cells by eliminating ROS and relieve the level of ER stress [64]. Keap1 is an adaptor of CUL3-ubiquitin E3 ligase [65]. Under quiescent conditions, the transcription factor Nrf2 interacts with the actin-anchored protein Keap1, largely localized in the cytoplasm. This quenching interaction results in proteasomal degradation of Nrf2 and maintains low basal expression of Nrf2-regulated genes. Once chemical signals imparted by oxidative and electrophilic molecules are recognized, the E3 ligase activity of the ubiquitin Keap1–CUL3 complex is decreased, and Nrf2 is dissociated from the binding of Keap1, escapes proteasomal degradation, and translocates into the nucleus where it transactivates the expression of several dozen cytoprotective genes including HO-1 and NQO1, which enhances cell survival [66,67]. However, the underlying mechanisms regarding the regulation of Keap1/Nrf2/ARE pathways during ER stress as well as cancer chemoresistance have not been fully elucidated. Therefore, we first explored the effect of DOX on the Keap1/Nrf2 antioxidant pathway and found that under the treatment of DOX with gradient concentration and time, the expression of Nrf2 gradually increased, while the expression of Keap1 gradually decreased, suggesting that the Keap1/Nrf2 pathway was activated under the treatment of DOX. Furthermore, we found that EDEM1 can up-regulate the expression of Nrf2, and this regulatory process is realized by down-regulating its ubiquitination level. At the same time, we demonstrated that EDEM1 can promote Nrf2 entry into the nucleus and activate the ARE transcription element, up-regulating the expression of downstream antioxidant molecules such as SOD1. Since Keap1 can bind Nrf2 and promote its ubiquitination degradation, we hypothesize whether EDEM1 can play a role in Nrf2 activation by interfering with the binding of Keap1 and Nrf2. Therefore, we conducted IP and co-IP experiments and found that EDEM1 can bind to Keap1, but not Nrf2. Combined with the previous results, we hypothesized that the regulatory effect of EDEM1 on the Keap1/Nrf2 pathway was achieved through competitive binding of Keap1 so that Keap1 could not bind and degrade Nrf2. Previous studies reported that Nrf2 mainly binds to the DGR domain of Keap1, and the DGR domain of Keap1 can also directly or indirectly bind to other proteins [68]. Therefore, we generated a series of truncated mutants of Keap1, further proving that EDEM1 is bound with the DGR domain of Keap1, which verified our hypothesis of competitive binding.

Ferroptosis is an iron-dependent form of cell death characterized by extensive lipid peroxidation [69]. Studies have shown that chemotherapy, such as treatment with DOX, can induce ferroptosis in cancer cells through mechanisms involving excessive ROS production and iron dysregulation [70]. Based on the well-established role of Nrf2 activation in suppressing ferroptosis primarily achieved through the up-regulation of antioxidant genes [71] and our findings, we proposed that EDEM1 could alleviate oxidative stress and promote cancer cell survival at both the ER stress and ferroptosis levels. First, EDEM1 directly alleviates DOX-induced ferroptosis by activating the Keap1/Nrf2 antioxidant pathway, which enhances the expression of antioxidant genes, thereby reducing ROS levels and preventing lipid peroxidation, a hallmark of ferroptosis. Second, EDEM1 indirectly mitigates ferroptosis by alleviating ER stress through its role in ERAD. By enhancing the degradation of misfolded proteins, EDEM1 reduces ER luminal ROS production, further mitigating lipid peroxidation and decreasing the production of predisposing factors for ferroptosis. Therefore, EDEM1 exerts a protective effect against ferroptosis through dual mechanisms: it activates the Keap1/Nrf2 antioxidant pathway to directly counteract oxidative stress and lipid peroxidation; concurrently, it enhances ERAD to reduce ROS production from misfolded protein accumulation. These findings highlight the multifaceted role of EDEM1 in regulating cell death pathways, and its impact on ferroptosis warrants further investigation in the future.

miRNAs are small noncoding RNAs approximately 22 nt in length, generated by Dicer from precursors with hairpin secondary structures [72], which primarily inhibit the posttranscriptional expression of target mRNAs by interacting with the 3′UTR. Importantly, the expression profiles of miRNAs in human cancers are closely associated with cancer detection, stage, progression, and therapeutic response [73]. For instance, tumor-derived exosomal miR-934 has been shown to induce macrophage M2 polarization, thereby promoting liver metastasis of colorectal cancer via down-regulating phosphatase and tensin homolog deleted on chromosome ten (PTEN) expression and activating the PI3K/AKT signaling pathway [74]. Additionally, miR-99-5p has been identified as a key regulator of endocrine and HER2-targeted therapy resistance by modulating ESR1 expression [75]. Given these multifaceted roles of miRNAs in cancer biology and therapy, we specifically focused on miRNAs as potential upstream regulators of EDEM1 expression and function. By integrating data from multiple databases, we identified several miRNAs that may play a notable role in regulating EDEM1. Our study demonstrated that miR-32-5p could regulate the expression of EDEM1, with 2 binding sites identified on the 3′UTR of EDEM. We further explored the biological functions of miR-32-5p in breast cancer and conducted rescue experiments to elucidate its regulatory effect on EDEM1. Our results showed that miR-32-5p overexpression led to suppressed EDEM1 level and promoted NRF2 degradation, which is associated with impaired antioxidant effects and increased sensitivity to chemotherapeutics. Conversely, coexpression of EDEM1 could rescue the inhibited biological functions caused by miR-32-5p, further highlighting the importance of miR-32-5p as a potential therapeutic target to overcome EDEM1-mediated chemoresistance. In the future, more efforts are needed to comprehensively explore its regulatory mechanism and clinical application in breast cancer.

Screening out prognostic indicators of breast cancer is also one of the significant focuses of current research. Previous studies have shown that the establishment of predictive models of ER stress-related molecular expression characteristics had a better predictive effect on the prognosis of gastric adenocarcinoma patients [76]. Meanwhile, several studies proved EDEM1 as a prognostic indicator in female lung adenocarcinoma [28] and colorectal cancer [77]. Considering that the clinical relevance and prognostic value of EDEM1 in breast cancer have not been fully investigated, we further explored the prognostic value of EDEM1. Through analyzing the IHC scores of 131 patients with breast cancer, we found that the high EDEM1 expression was associated with poor prognosis of breast cancer patients. At the same time, we found that the IHC scores of TNBC patients were prominently higher than those of non-TNBC patients. Therefore, we further analyzed EDEM1 expression in 76 TNBC patients and found that high EDEM1 expression is an independent risk factor for poor OS in TNBC. These results suggest that EDEM1 can be a potential prognostic marker. Besides, current research is increasingly focusing on the use of plant extracts or ER stress inducers to address challenges related to drug resistance and cancer metastasis. For instance, studies have shown that ononin can inhibit breast cancer progression by targeting the mitogen-activated protein kinase (MAPK) pathway [78]. In melanoma cells resistant to vemurafenib (BRAFV600E kinase inhibitor), combination treatment with TG, an ER stress inducer, markedly augmented apoptotic induction and restored drug efficacy [79]. Inspired by these findings, we speculate that modulating ER stress signaling and disrupting tumor cell adaptive responses through the use of plant-derived compounds, EDEM1 inhibitors, or ER stress inducers may represent a novel strategy to overcome chemoresistance in TNBC. However, further preclinical and clinical investigations are needed to validate its efficacy and safety.

## Conclusion

In conclusion, our study demonstrated that EDEM1 promoted TNBC progression and chemoresistance in vitro and in vivo. As a member of the ERAD process, EDEM1 accelerated ERAD and alleviated oxidative stress through activating the Keap1/Nrf2/ARE signaling pathway to maintain ER homeostasis. The expression and function of EDEM1 were down-regulated by upstream miR-32-5p. Moreover, high EDEM1 expression was associated with poor prognosis in breast cancer patients. Taken together, our findings identified EDEM1 as a potential therapeutic target and prognostic indicator for breast cancer, especially TNBC treatment in the future.

## Materials and Methods

### Plasmids, miRNA mimics, siRNA, and reagents

CD3-δ-YFP was purchased from Addgene (#12499, Massachusetts, USA), Flag-EDEM1 (human) was from WZ Biosciences (CH889882, Shandong, China). Human Keap1 cDNA sequence was used as a template, and Keap1 WT and Keap1 mut (ΔBTB, ΔIVR, and ΔDGR) were cloned into pCMV-C-Myc vectors (Beyotime, D2672, Shanghai, China). Vectors pCMV-C-HA and HA-Ub were kind gifts from Dianwen Han (Shandong University, Jinan, China). The human Nrf2 cDNA sequence was used as a template and was cloned into both pCMV-C-Myc and pCMV-C-HA vectors. Plasmids pRL-TK (E2241) and pmirGLO (E1330) were all from Promega (Madison, Wisconsin, USA). Hsa-miR-32-5p mimic, EDEM1 siRNA, and negative control (NC) were purchased from GenePharma (Shanghai, China). The sequence is shown in Table S7. Transfections were performed using Lipofectamine 2000 (Invitrogen, 11668019, Carlsbad, California, USA) according to the manufacturer’s protocol. Other reagents and their sources were indicated as follows: TM (11089-65-9, Sigma-Aldrich, Missouri, USA) or TG (67526-95-8, Sigma-Aldrich, Missouri, USA).

### Antibodies

The anti-EDEM1 rabbit polyclonal (26226-1-AP, 1:1,000 for WB, 1:200 for IHC), anti-Nrf2 rabbit polyclonal (16396-1-AP, 1:1,000 for WB, 1:200 for IHC and IF), anti-β-actin mouse monoclonal (66009-1-Ig, 1:1,000), and anti-Ki67 rabbit polyclonal (27309-1-AP, 1:500) antibodies were purchased from Proteintech (Illinois, USA). The anti-GRP78 rabbit polyclonal (AF0171, 1:1,000) antibody was obtained from Beyotime Biotechnology (Shanghai, China). The anti-Keap1 mouse monoclonal (sc-365626, 1:200) antibody was from Santa Cruz Biotechnology (California, USA). The anti-Flag rabbit monoclonal (F2555, 1:1,000), and anti-HA rabbit polyclonal (H6908, 1:1,000) antibodies were from Sigma-Aldrich (Missouri, USA). The anti-Myc (A190-105A, 1:1,000) antibody was from Bethyl Laboratories (Texas, USA).

### Cell culture

The human immortalized breast epithelial cell line (MCF-10A), human breast cancer cell lines (MCF-7, T47D, ZR75-1, SKBR3, MDA-MB-453, MDA-MB-231, MDA-MB-468, and HS578T), and HEK-293T cells were purchased from the American Type Culture Collection (Manassas, Virginia, USA). Cells were cultured in Dulbecco's modified Eagle's medium (DMEM) (Macgene, CM10017, Beijing, China) supplemented with 10% fetal bovine serum (FBS) (Gibco, Carlsbad, California, USA), and 1% penicillin and streptomycin (Macgene, CC004, China, Beijing, China). All these cells were cultured in an incubator with 5% CO_2_ at 37 °C under saturated humidity.

### Apoptosis assay

Cells were treated with TM (5 μg/ml), TG (1 μM), or DOX (1 μM) for 24 h. Then, cells treated with TM and TG were collected and analyzed following the instructions of BD Pharmingen PE Annexin V Apoptosis Detection Kit (BD Bioscience, 559763, New Jersey, USA). Cells treated with DOX were collected and analyzed following the instructions of the Annexin V-FITC/7-AAD Apoptosis Detection Kit (bjbalb, HR8280, Beijing, China). Briefly, cells were washed 2 times in PBS, suspended in 1 × Binding Buffer, and incubated with 7-aminoactinomycin D (7-AAD)-peridnine chlorophyll protein (PerCP) and Annexin V-phycoerythrin (PE)/fluorescein isothiocyanate (FITC) at room temperature for 20 min. Then, the apoptosis rate of cultured cells was detected by a FACSCalibur flow cytometer (BD Bioscience, New Jersey, USA) and analyzed by FlowJo_v10.6.2 software. Apoptosis rate = early apoptosis rate (Annexin V+/7-AAD-, Q3) + late apoptosis rate (Annexin V+/7-AAD+, Q2).

### ROS analysis

The ROS levels of cultured cells were detected by the Reactive Oxygen Species Assay Kit (Solarbio, CA1410, Beijing, China). Cells were collected, washed 3 times with PBS, incubated with DCFDA at a final concentration of 10 μM in FBS-free DMEM for 30 min at 37 °C in the dark, and then washed 3 times with cold PBS. Then, cells were collected and suspended with 300 μl of cold PBS. The ROS levels were detected by a FACSCalibur flow cytometer (BD Bioscience, New Jersey, USA) and analyzed by FlowJo_v10.6.2 software.

### Nuclear and cytoplasmic extraction

Nuclear and cytoplasmic proteins of treated cells were obtained using the Nuclear and Cytoplasmic Protein Extraction Kit (Beyotime, P0027, Shanghai, China) according to the manufacturer’s instructions. Briefly, cells were washed in ice-cold PBS and resuspended using Cytoplasmic Protein Extraction Buffer A with 1 mM phenylmethylsulfonyl fluoride (PMSF). The cell suspension was vortexed violently at maximum speed for 5 s and sat on ice for 15 min. Then, cell suspension was added with Cytoplasmic Protein Extraction Buffer B, vortexed violently at maximum speed for 5 s, and sat on ice for 1 min. After centrifugation at 12,000 *g* for 5 min at 4 °C, the supernatant was aspirated as cytoplasmic fraction. To obtain the nuclear fraction, the cell pellet was suspended using a Nuclear Protein Extraction Buffer with 1 mM PMSF. Then, cell suspension was alternately vortexed for 15 s and on an ice bath for 2 min for a total of 30 min. Lysates were centrifuged at 12,000 *g* for 10 min at 4 °C, and the supernatant was collected as a nuclear fraction. The concentration of protein was analyzed using a BCA protein assay kit (Beyotime, P0010, Shanghai, China).

### Dual-luciferase reporter assay

ARE luciferase Reporter plasmid was obtained from Yeasen Biotech Co., Ltd (11548ES03, Shanghai, China). The EDEM1 3′UTR including the miR-32-5p binding sites were cloned into the pmirGLO Dual-Luciferase miRNA Target Expression Vector (Promega, Madison, Wisconsin, USA) to form the reporter vector. There were 2 predicted binding sites of miR-32-5p on the EDEM1 3′UTR region and point mutations of 2 sites were specifically synthesized and inserted into the pmirGLO vector. A total of 1.5 × 10^5^ cells were seeded in 48-well plates. On the next day, 100 μM NC and 100 μM si-EDEM1 were cotransfected with EDEM1-Wild type or EDEM1-Mutant reporter vectors into HEK-293T cells. After 48 h, the firefly and the Renilla activity was measured using the Dual-Luciferase Reporter Assay System (Promega, Madison, Wisconsin, USA). For each sample, relative firefly luciferase activity was normalized to Renilla luciferase activity as a control of transfection efficiency.

### Immunofluorescence

Transfected cells were cultured under indicated treatments on cell climbing pieces, washed 3 times with PBS, fixed with 4% Paraformaldehyde (PFA) Fix Solution at room temperature for 20 min, and permeabilized with 0.2% Triton X-100 for 15 min. Then, cells were washed 3 times with PBS and blocked with 10% goat serum for 1 h. After blocking, cells were incubated with mentioned primary antibodies at 4 °C overnight and FITC- or Rho-conjugated secondary antibodies, then dyed by 4′,6-diamidino-2-phenylindole (DAPI; Beyotime, C1005, Shanghai, China), and observed and imaged under an inverted fluorescence microscope (ZEISS, Jena, Germany).

### Tumor xenograft mouse models

For the in vivo tumorigenicity assay, female Balb/c mice (4 to 5 weeks) were purchased from GemPharmatech Co., Ltd. (Nanjing, China) and housed under standard conditions at the animal care facility. Tumor cells (1 × 10^7^ MDA-MB-231 cells) stably expressing EDEM1 or control vector were orthotopically injected directly into the mammary fat pads of mice (*n* = 10 per group) in 200 μl of sterile PBS. When the volume of subcutaneous tumors reached an average of 50 mm^3^, the nude mice in each group were divided randomly and evenly into 2 subgroups (*n* = 5 per subgroup). In the chemotherapy subgroup, mice were treated with DOX (3 mg per kg) in 100 μl by intravenous injection every 3 days for a total of 7 times, while mice in the control subgroup were given equal volumes of PBS. The tumor volume was measured every 4 days and calculated according to the following formula: volume = 0.5 × length × (width)^2^. At day 35 post-cancer cell implantation, the animals were sacrificed and tumor weights and volumes were recorded and analyzed. The excised tumors were then fixed in formalin for hematoxylin and eosin (H&E) staining and IHC analysis.

As for lung metastasis models, 5 × 10^5^ of the abovementioned cells were intravenously injected into the tail vein of nude mice. One month after injection, the animals were euthanized, the lungs were captured, and the number of metastatic foci was counted. Then lung tissues were fixed with formalin, embedded in paraffin, and cut into slices for H&E staining. All animal experiments were approved by the Shandong University Animal Care and Use Committee.

### Clinical samples

For this study, we collected 25 paired breast cancer tissue samples and adjacent normal tissues from patients who had been diagnosed with primary breast cancer by pathological assessment of tissues and had undergone surgeries at the Qilu Hospital of Shandong University. Tissue samples were used for quantification of EDEM1 expression. We also collected complete prognostic information of 131 breast cancer patients at the Qilu Hospital of Shandong University from March 2007 to January 2017 for Kaplan–Meier survival analysis and Cox regression analysis. All samples were collected from participants with informed consent, and all related procedures were approved by internal review and ethics boards of the Ethical Committee of Qilu Hospital of Shandong University.

### H&E staining and IHC analysis

Tissues were formalin-fixed, paraffin-embedded, and then sectioned at 4-μm thickness for H&E staining and IHC staining. As for H&E staining, the slides were then deparaffinized with xylene and hydrated using gradient ethanol. After staining with H&E in turn, the sections were dehydrated using gradient ethanol, vitrified by dimethyl benzene, and deposited in Permount TM Mounting Medium (Solarbio, 96949-21-2, Beijing, China) for image acquisition. IHC staining was conducted by a Universal 2-step detection kit (PV-9000, ZSGB-Bio, Beijing, China). After rehydration, antigen retrieval was performed in EDTA buffer (pH 9.0). After washing with PBST, the slides were incubated with Endogenous peroxidase blocking buffer (Reagent 1) and Reaction-enhancing buffer (Reagent 2) for 15 min at 37 °C, in turn. Then, the sections were incubated with primary antibodies at 4 °C overnight. On the next day, the slices were washed with PBST (0.1% Tween) and incubated with horseradish peroxidase-conjugated goat anti-mouse/rabbit antibody (Reagent 3) for 15 min at 37 °C. Then, the slides were stained with diaminobenzidine (DAB) (ZSGB-Bio, ZLI-9018, Beijing, China) and nuclei were counterstained with hematoxylin (Solarbio, G1080, Beijing, China). Then, the slides were dehydrated using gradient ethanol, vitrified by dimethylbenzene, and deposited in Permount TM Mounting Medium (Solarbio) for image acquisition.

Positive expression of EDEM1 (brown) was primarily detected in the cytoplasm. The staining scores were semiquantitatively evaluated according to both the intensity and proportion of EDEM1-positive cells at 400× magnification. The proportion score of positively stained tumor cells in sections was graded as follows: 0, <10%; 1, 10% to 25%; 2, 25% to 50%; 3, 50% to 75%; and 4, >75%. The cells at each staining intensity were recorded as staining intensity score: 0, no staining; 1, light brown; 2, brown; and 3, dark brown. The IHC score was calculated as follows: IHC score = proportion score × staining intensity score. Using this method, the expression of EDEM1 was evaluated using the IHC score and scored as 0, 1, 2, 3, 4, 6, 8, 9, or 12. The expression state of EDEM1 was classified into 4 grades: negative, IHC score ≤ 3; weak, IHC score >3 and ≤ 6; moderate, IHC > 6 and ≤ 9; and strong, IHC > 9. With a cutoff point of 7 calculated by the receiver operating characteristic (ROC) curves, EDEM1 expression was divided into a low-expression group (negative and weak groups) and a high-expression group (moderate and strong groups).

### Statistical analysis

All experiments were independently repeated at least 3 times. Experiment data analysis was performed using GraphPad Prism 8.0.1 software (La Jolla, California, USA). Clinical data analysis was performed using SPSS software (Version 22.0). The ROC curve was used to assess the diagnostic value. For Kaplan–Meier analyses of breast cancer patients, a log-rank test was used to calculate statistical differences in survival curves. The unpaired 2-tailed Student’s *t* test and chi-square test were used to calculate the statistical significance between the 2 groups. Analysis of variance test was used to compare the mean values of 2 or more samples. The effects of the clinical features on OS and DFS of breast cancer patients were determined through univariate and multivariate Cox regression analyses. *P* < 0.05 was considered significant in all statistical tests.

## Ethical Approval

All animal investigations were approved by the Shandong University Animal Care and Use Committee. The human study was approved by the Ethical Committee of Qilu Hospital of Shandong University. The informed consents for participation in this study from all patients were obtained.

## Data Availability

The data and material are available by contacting the corresponding author upon reasonable request.

## Supplementary Materials

Supplementary 1 Experimental section Figs. S1 to S10 Tables S1 to S8
