# Supplementary material for: EDEM1 Inhibits Endoplasmic Reticulum Stress to Induce Doxorubicin Resistance through Accelerating ERAD and Activating Keap1/Nrf2 Antioxidant Pathway in Triple-Negative Breast Cancer
Source: Research (Wash D C). 2025 Jul 29;8:0797. doi: 10.34133/research.0797 (PMC12305169; doi:10.34133/research.0797)
Supplement: Supplementary 1 — Experimental section Figs. S1 to S10 Tables S1 to S8 [file research.0797.f1.pdf]

Supporting Information

EDEM1 Inhibits ER Stress to Induce Doxorubicin Resistance through Accelerating ERAD and Activating Keap1/Nrf2 Antioxidant Pathway in Triple-Negative Breast Cancer.

Yajie Wang[1, #], Yiran Liang[1, #], Dan Luo[1, #], Fangzhou Ye[1], Yuhan Jin[1], Lei Wang[1], Yaming Li[1], Dianwen Han[1], Zekun Wang[1], Bing Chen[2], Wenjing Zhao[2], Lijuan Wang[2], and Qifeng Yang[1,2,3, *]

[1]Department of Breast Surgery, General Surgery, Qilu Hospital of Shandong University, Jinan, Shandong, 250012, China

[2]Biological Resource Center, Qilu Hospital of Shandong University, Jinan, Shandong, 250012, P.R. China

[3]Research Institute of Breast Cancer, Shandong University, Jinan, Shandong, 250012, P.R. China

[#]These authors contributed equally to this work.

[*]**Correspondence:** Qifeng Yang

Qilu Hospital of Shandong University, Wenhua Xi Road No. 107, Jinan 250012, Shandong, China; Tel: 86-18560085168; E-mail: qifengy_sdu@163.com

Supplementary Experimental Section

Cell proliferation assays and cell viability assay: Cell proliferation and cell viability were measured using MTT assays. Briefly, suspended cells (1,500 for proliferation assay, 3,000 for cell viability assay) cultured in DMEM medium were seeded in 96-well plates. 20 μL of MTT was added to each well and incubated for 6 h at 37°C. Viable cells were stained by 3-(4,5-dimethylthiazol-2-yl)-2,5-diphenyltetrazolium bromide (MTT) (Solarbio, M8180, Beijing, China). 100 μL DMSO per well was added and the absorbance at 490 nm was then measured at the indicated time points.

EdU assay: EdU assay was used to detect cell proliferation using a Cell-Light™ EdU Apollo567 In Vitro Kit (RiboBio, C10310-1, Guangzhou, China) following the manufacturer's instructions. Briefly, 2×10^4 transfected cells were suspended in DMEM containing 10% FBS and then seeded on 96-well plates for 24 h. On the next day, cells were incubated with 100 μl of 50 μM EdU for 2 h at 37°C. Then, cells were fixed with 4% PFA and stained with 1× Apollo solution and 1× Hoechst 33342 solution. Finally, cells were visualized under the fluorescence microscope (Zeiss, Jena, Germany).

Cell migration and invasion assays: Migration and invasion assays were performed using transwell assay. $6\text{-}8\times10^4$ transfected cells were suspended in 200 μL FBS-free DMEM and were added to the upper chamber. The lower chamber was filled with 700 μL of DMEM containing 20% FBS. In the invasion assay, the 8-μm polycarbonate membrane of the transwell chamber (Corning, New York, USA) was coated with 60 μl DMEM-diluted ice-cold Matrigel (Corning, 356232, New York, USA) before adding the cell suspension. After cell culture for 24-48h, cells on the lower compartment were

washed with PBS, fixed with methanol, and stained with 0.2 % crystal violet for 20 min, then imaged and counted.

Wound healing assays: 3×10^5 transfected cells were seeded in 24-well plated and reached 100 % confluence. Then a linear wound was generated on cell monolayer with sterile 10 μL pipette tips. Floating cells were then removed and the cultures were maintained in DMEM without FBS. The progression of migration was imaged with an inverted microscope at 0 h and 48 h after scraping. The healing indexes representing the migration ability of cancer cells were calculated using the following formula: $(S_0-S_{48})/S_0 \times 100\%$. S_0 and S_{48} respectively represent the blank area at 0 h and 48 h after the scape.

Quantitative real-time PCR: RNAiso Plus (Total RNA extraction reagent) (Takara, 9108, Kyoto, Japan) was used to extract RNA from breast cancer cell lines, maintained cells under different conditions, and cancer tissues according to the manufacturer's protocol. 1 μg RNA was reverse transcribed by the PrimeScript reverse transcriptase (RT) reagent kit (Takara, RR037B, Kyoto, Japan). Mir-X miRNA First-Strand Synthesis Kit (Takara, 638315, Kyoto, Japan) was used to reverse miRNA. TB Green™ Advantage ® qPCR Premix (Takara, 639676, Kyoto, Japan) was used to conduct qRT-PCR. Actin was used as the internal reference for mRNA, while U6 was for miRNA. The qRT-PCR primers were showed in Table S8.

Western blot: Cells were lysed in cell lysis buffer (Beyotime, P0013, Shanghai, China) containing 1 mM NaF and 1 mM PMSF for 2 h on ice then centrifuged at 4 °C at 12,000 rpm for 30 min. The proteins were quantified via a BCA protein assay kit (Beyotime,

P0010, Shanghai, China) and boiled at 95℃ after being mixed with SDS loading buffer. Proteins were separated by 10% SDS-PAGE and electro-transferred onto 0.22 μm polyvinylidene difluoride (PVDF) membranes (Millipore, Billerica, Massachusetts, USA), then blocked with 5 % skimming milk at room temperate for at least 1 h. The membranes were incubated with primary antibodies above mentioned at 4 °C overnight then washed 3 times with TBST and incubated with secondary antibodies (1:3000) (ZSGB-BIO, Beijing, China) at room temperature for 2h. Finally, an Enhanced chemiluminescence (ECL) kit (Vazyme, E423-01, Nanjing, China) was used to visualize the bands.

TUNEL assay: To detect apoptosis in tumor tissue, TUNEL assay was performed using One-step TUNEL cell apoptosis detection kit (Beyotime, C1086, Shanghai, China) according the manufacturer's instruction. The slides were deparaffinized with xylene and hydrated using gradient ethanol. After adding 20μg/ml protease K without DNase at 20-37℃ for 15-30 minutes, the slides were washed with PBS for 3 times. TUNEL test solution was prepared according the manufacturer's instruction. Then slides were incubated by TUNEL test solution at 37℃ for 60 minutes away from light, wash with PBS for 3 times, then dyed by DAPI (Beyotime, C1005, Shanghai, China), observed and imaged under an inverted fluorescence microscope (ZEISS, Jena, Germany).

Supplementary Figures and Legends

Figure S1. EDEM1 overexpression promotes the progression of TNBC cells in vitro.

A) Differential expression of EDEM1 between normal and tumor tissues in TCGA and Metabric database. B) Relative mRNA fold change of EDEM1 in MDA-MB-231 and MDA-MB-468 cells transfected with pENTER and Flag-EDEM1 plasmids. C)-D) MTT (C), and EdU (D) assays for cell proliferation ability of control and EDEM1-overexpressing MDA-MB-231 and MDA-MB-468 cells. Scale bar = 100 μm. E) Flow cytometry analysis of cell cycle distributions in control and EDEM1-overexpressing MDA-MB-231 and MDA-MB-468 cells. F) Transwell assays for migration and invasion abilities of control and EDEM1-overexpressing MDA-MB-231 and MDA-MB-468 cells. Scale bar = 100 μm. G) Wound healing assays for migration abilities of control and EDEM1-overexpressing MDA-MB-231 and MDA-MB-468 cells. Scale bar = 200 μm. (ns, no significance, *$P < 0.05$, **$P < 0.01$, ***$P < 0.001$)

Figure S2. EDEM1 knockdown inhibits the progression of TNBC cells in vitro.

A) Relative mRNA fold change of EDEM1 in MDA-MB-231 and MDA-MB-468 cells transfected with si-NC and si-EDEM1 mimics. B)-C) MTT (B), and EdU (C) assays for cell proliferation abilities of control and EDEM1-knockdown MDA-MB-231 and MDA-MB-468 cells. Scale bar = 100 μm. D) Flow cytometry analysis of cell cycle distributions in control and EDEM1-knockdown MDA-MB-231 and MDA-MB-468 cells. E) Transwell assays for migration and invasion abilities of control and EDEM1-knockdown MDA-MB-231 and MDA-MB-468 cells. Scale bar = 100 μm. F) Wound

healing assays for migration abilities of control and EDEM1- knockdown MDA-MB-231 and MDA-MB-468 cells. Scale bar = 200 μm. (ns, no significance, *P < 0.05, **P < 0.01, ***P < 0.001)

Figure S3. EDEM1 knockdown inhibits the progression of DOX-resistant TNBC cells.

A-B) The RNA (A) and protein (B) expression of EDEM1 in MDA-MB-231 and 231/DOX cells. C) Relative mRNA fold change of EDEM1 in 231/DOX cells transfected with si-NC and si-EDEM1 mimics. D) MTT assays for cell proliferation abilities of control and EDEM1-knockdown 231/DOX cells. E) EdU assays for cell proliferation abilities of control and EDEM1-knockdown 231/DOX cells. Scale bar = 100 μm. F) Transwell assays for migration and invasion abilities of control and EDEM1-knockdown 231/DOX cells. Scale bar = 100 μm. G) Wound healing assays for migration abilities of control and EDEM1-knockdown 231/DOX cells. Scale bar = 200 μm. H) Flow cytometry analysis of apoptosis rates in control and EDEM1-knockdown 231/DOX cells. (*P < 0.05, **P < 0.01, ***P < 0.001)

Figure S4. EDEM1 plays a vital role in ER stress-induced apoptosis and slightly activates the UPR pathway of TNBC cells in vitro.

A) Flow cytometry analysis of apoptosis rates in control and EDEM1-knockdown 231/DOX cells pre-treated with TM (5 μg/ml) and TG (1 μM) for 24 h. B) Relative mRNA fold change of sXBP1, ATF4, CHOP, and ATF6 in MDA-MB-231 and MDA-MB-468 cells transfected with pENTER and Flag-EDEM1 plasmids. (ns, no

significance, *P < 0.05, **P < 0.01, ***P < 0.001)

Figure S5. EDEM1 attenuates autophagy of TNBC.

A) MDC staining of control and EDEM1-overexpressing MDA-MB-231 cells pre-treated with starvation for 6 h and MDA-MB-468 cells pre-treated with starvation for 20 h. Scale bar = 50 μm. B) MDC staining of control and EDEM1-knockdown MDA-MB-231 cells pre-treated with starvation for 6 h and MDA-MB-468 cells pre-treated with starvation for 20 h. Scale bar = 50 μm. C) Immunofluorescence staining of control and EDEM1-overexpressing MDA-MB-231 cells pre-treated with starvation for 6 h (left) and MDA-MB-468 cells pre-treated with starvation for 20 h (right). Scale bar = 50 μm. D) Immunofluorescence staining of control and EDEM1-knockdown MDA-MB-231 cells pre-treated with starvation for 6 h (left) and MDA-MB-468 cells pre-treated with starvation for 20 h (right). Scale bar = 50 μm.

Figure S6. Predicted miRNAs screening.

A) The correlation between predicted miRNAs and patients' prognosis using Kaplan-Meier plotter (https://kmplot.com/analysis/). B) Relative mRNA fold change of miR-32-5p in human normal mammary epithelial cells (MCF-10A) and breast cancer cell lines (MCF-7, T47D, ZR75-1, SKBR3, MDA-MB-453, MDA-MB-468, MDA-MB-231, HS578T). C) Correlation analysis of the expression between miR-32-5p and EDEM1 in human normal mammary epithelial cells (MCF-10A) and breast cancer cell lines (MCF-7, T47D, ZR75-1, SKBR3, MDA-MB-453, MDA-MB-468, MDA-MB-231, HS578T). D) Prediction of expression correlation between miR-32-5p and

EDEM1 by StarBase (https://rnasysu.com/encori/panMirCoExp.php).

Figure S7. miR-32-5p inhibits TNBC progression and chemoresistance.

A) Relative mRNA fold change of miR-32-5p in MDA-MB-231 and MDA-MB-468 cells. B) MTT assays for cell proliferation abilities of MDA-MB-231 and MDA-MB-468 cells transfected with NC and miR-32-5p mimics. C) EdU assays for cell proliferation abilities of MDA-MB-231 and MDA-MB-468 cells transfected with NC and miR-32-5p mimics. Scale bar = 100 μm. D) Transwell assays for migration and invasion abilities of MDA-MB-231 and MDA-MB-468 cells transfected with NC and miR-32-5p mimics. Scale bar = 100 μm. E) Wound healing assays for migration abilities of MDA-MB-231 and MDA-MB-468 cells transfected with NC and miR-32-5p mimics. Scale bar = 200 μm. F) Flow cytometry analysis of apoptosis rates of control and miR-32-5p overexpression MDA-MB-231 and MDA-MB-468 cells. G) Inhibitory curve of control and miR-32-5p overexpression MDA-MB-231 and MDA-MB-468 cells pre-treated with different concentrations of DOX for 48 h. (*$P < 0.05$, **$P < 0.01$, ***$P < 0.001$)

Figure S8. miR-32-5p suppresses TNBC progression through targeting EDEM1.

A) EdU assays for proliferation abilities of MDA-MB-231 and MDA-MB-468 cells transfected with indicated plasmids and mimics. Scale bar = 100 μm. B) Statistical graphs for EdU assays. C) Transwell assays for migration and invasion abilities of MDA-MB-231 and MDA-MB-468 cells transfected with indicated plasmids and mimics. Scale bar = 100 μm. D) Statistical graphs for migration assays. E) Statistical

graphs for invasion assays. F) Wound healing assays for proliferation abilities of MDA-MB-231 and MDA-MB-468 cells transfected with indicated plasmids and mimics. Scale bar = 200 μm. G) Statistical graphs for wound healing assays. (*P < 0.05, **P < 0.01, ***P < 0.001)

Figure S9. EDEM1 promotes TNBC progression and chemoresistance in vivo through targeting Nrf2. A) The TUNEL assay showed apoptotic cells in tumor tissues. Scale bar = 100 μm. B) Inhibitory curve of MDA-MB-231 and MDA-MB-468 cells transfected with indicated plasmids and mimics and pre-treated with different concentrations of DOX for 48 h. C) The image of tumors excised from all nude mice at day 35. D-E) Tumor volume (C) and tumor weight (D) are measured in MDA-MB-231 xenograft.

Figure S10. EDEM1 is correlated with poor clinical breast cancer prognosis.

A) ROC analysis of OS and DFS between high EDEM1 expression and low EDEM1 expression breast cancer patients. B) Log-rank test of OS and DFS between high EDEM1 expression and low EDEM1 expression breast cancer patients. C) Forest plot of independent risk factors in multivariate Cox analysis for OS and DFS of 131 breast cancer patients.

Figure S1

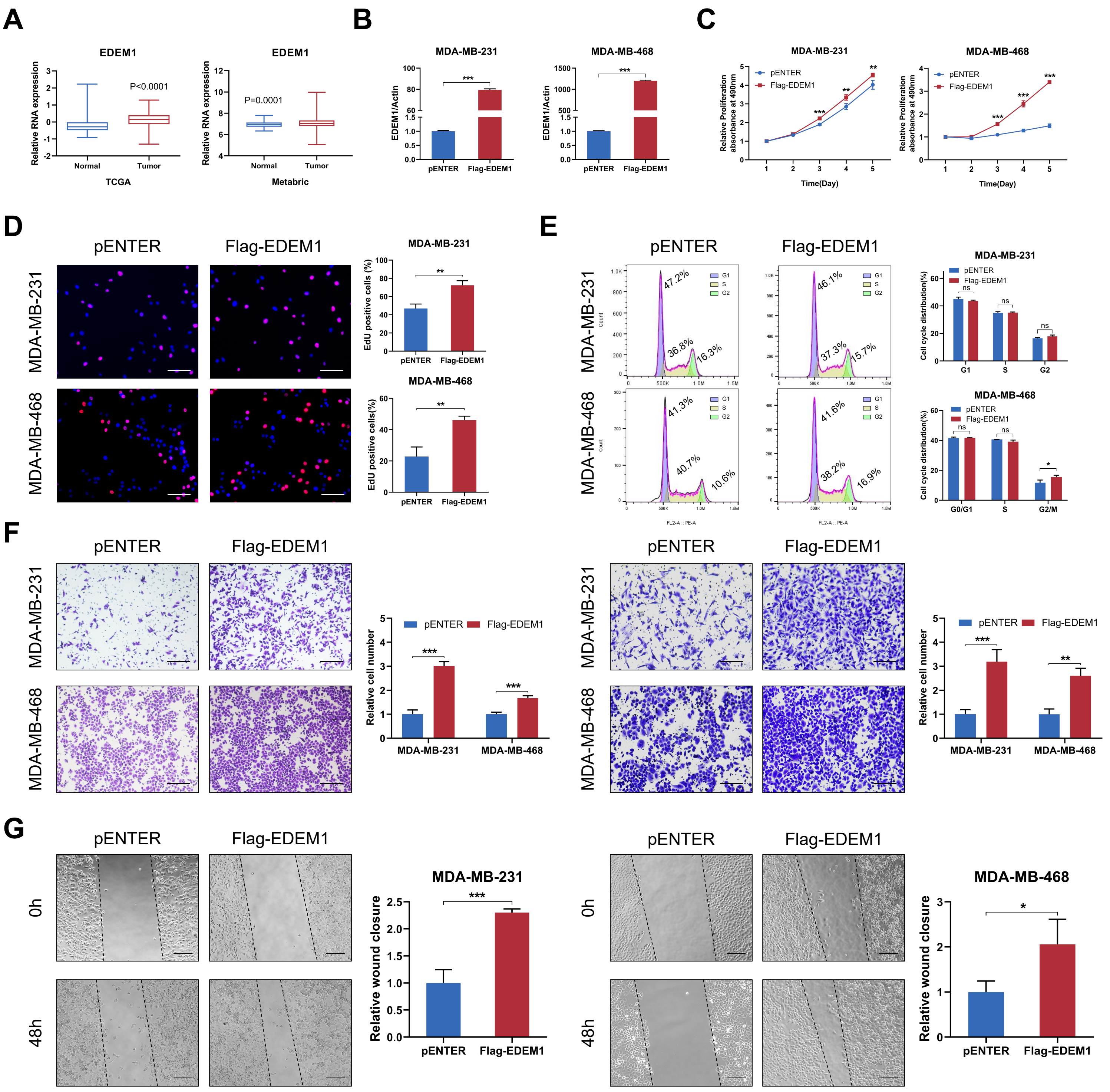
A
EDEM1
Relative RNA expression
P<0.0001
Normal
Tumor
TCGA
P=0.0001
Metabric
B
MDA-MB-231
MDA-MB-468
EDEM1/Actin
***
pENTER
Flag-EDEM1
C
Relative Proliferation absorbance at 490nm
**
***
Time(Day)
D
pENTER
Flag-EDEM1
MDA-MB-231
MDA-MB-468
EdU positive cells (%)
EdU positive cells(%)
E
47.2%
36.8%
16.3%
46.1%
37.3%
15.7%
41.3%
40.7%
10.6%
41.6%
38.2%
16.9%
G1
S
G2
Count
FL2-A :: PE-A
Cell cycle distribution(%)
ns
*
G0/G1
G2/M
F
Relative cell number
G
0h
48h
Relative wound closure

Figure S2

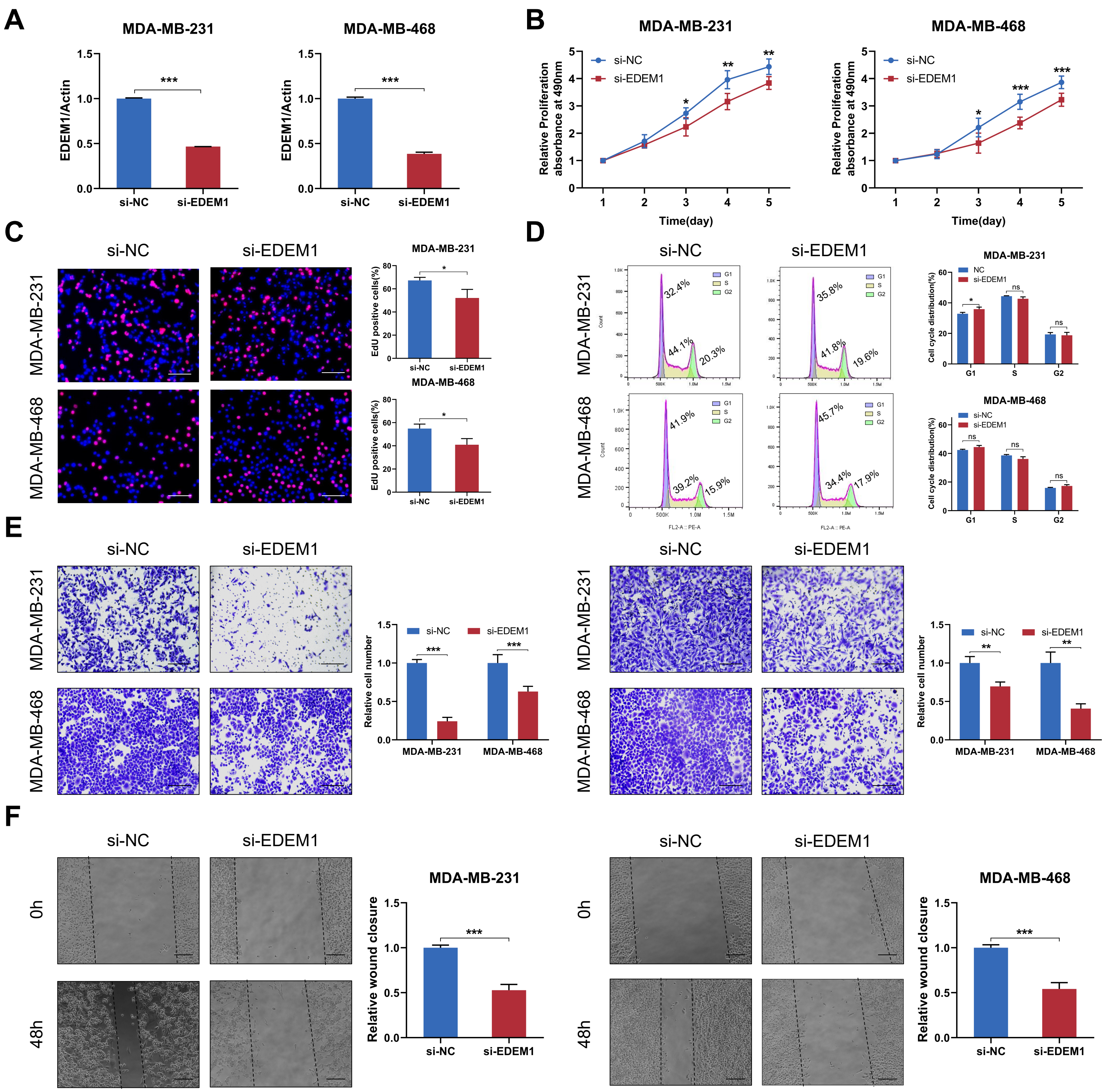
A
MDA-MB-231
MDA-MB-468
EDEM1/Actin
***
si-NC
si-EDEM1
B
Relative Proliferation absorbance at 490nm
Time(day)
C
EdU positive cells(%)
D
Cell cycle distribution(%)
NC
G1
S
G2
ns
32.4%
44.1%
20.3%
35.8%
41.8%
19.6%
41.9%
39.2%
15.9%
45.7%
34.4%
17.9%
FL2-A :: PE-A
Count
E
Relative cell number
F
0h
48h
Relative wound closure

Figure S3

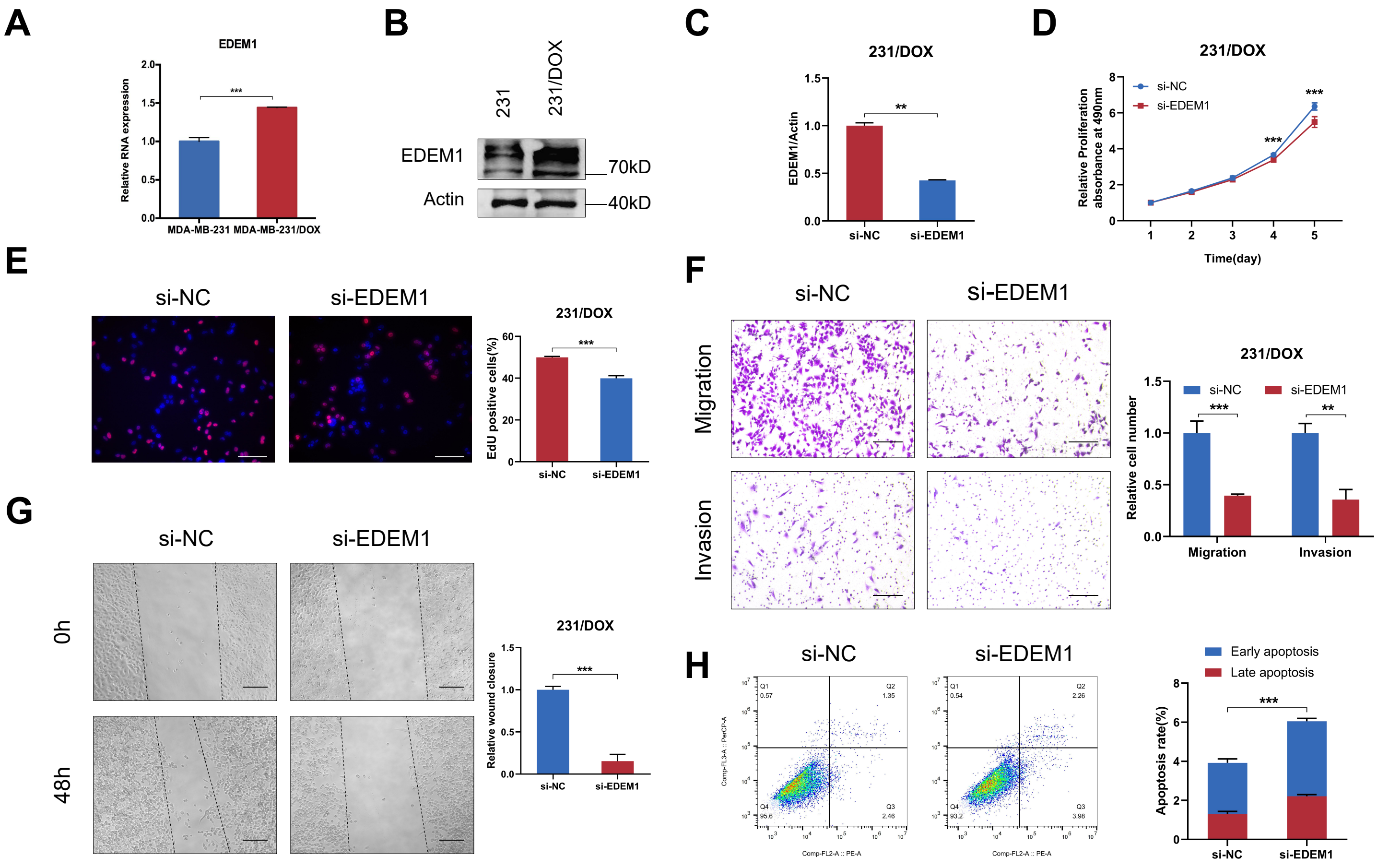
A
EDEM1
Relative RNA expression
***
MDA-MB-231
MDA-MB-231/DOX
B
231
231/DOX
EDEM1
70kD
Actin
40kD
C
231/DOX
EDEM1/Actin
**
si-NC
si-EDEM1
D
231/DOX
si-NC
si-EDEM1
Relative Proliferation absorbance at 490nm
***
***
Time(day)
E
si-NC
si-EDEM1
231/DOX
EdU positive cells(%)
***
si-NC
si-EDEM1
F
si-NC
si-EDEM1
Migration
Invasion
231/DOX
si-NC
si-EDEM1
Relative cell number
***
**
Migration
Invasion
G
si-NC
si-EDEM1
0h
48h
231/DOX
Relative wound closure
***
si-NC
si-EDEM1
H
si-NC
si-EDEM1
Q1 0.57
Q2 1.35
Q4 95.6
Q3 2.46
Q1 0.54
Q2 2.26
Q4 93.2
Q3 3.98
Comp-FL2-A :: PE-A
Comp-FL3-A :: PerCP-A
Early apoptosis
Late apoptosis
Apoptosis rate(%)
***
si-NC
si-EDEM1

Figure S4

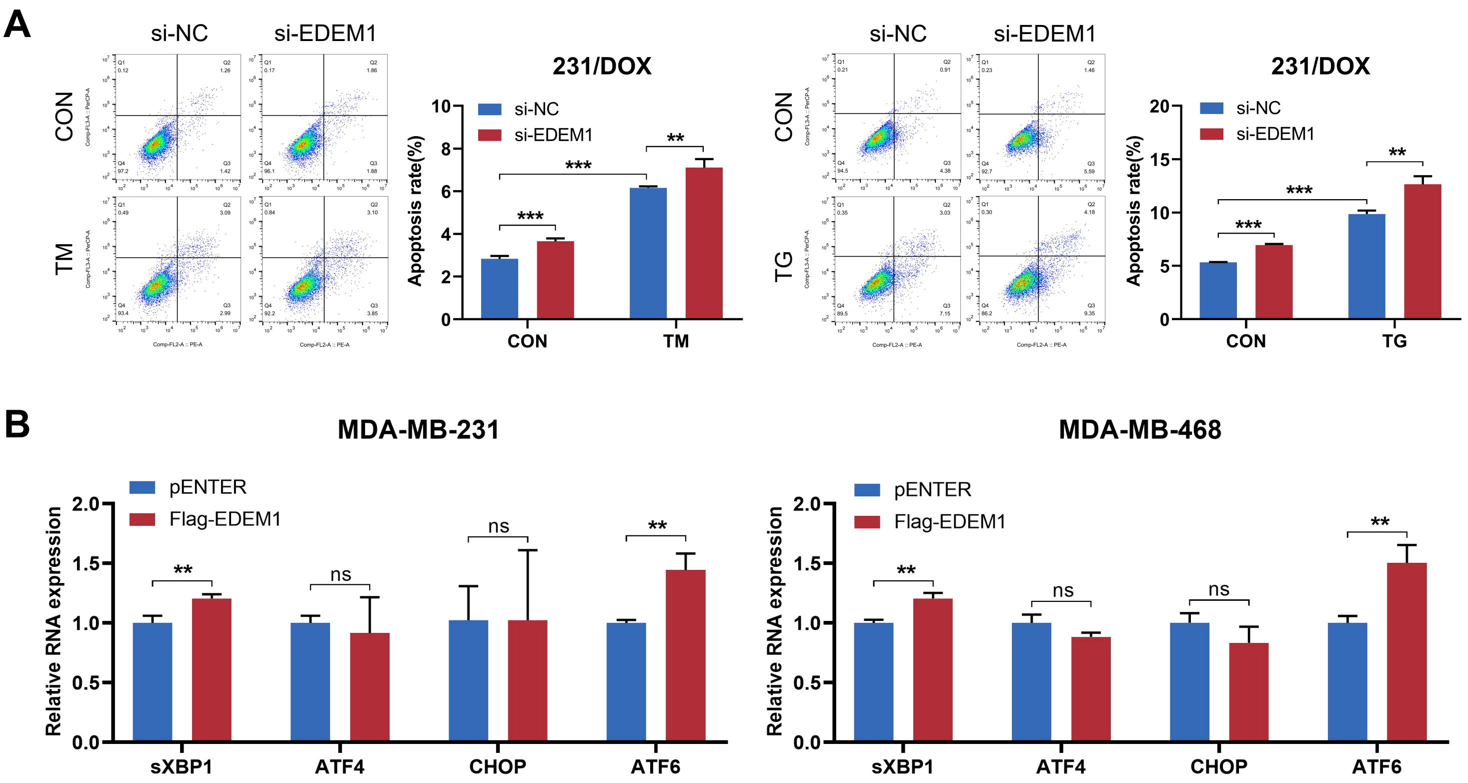
A
si-NC
si-EDEM1
CON
TM
231/DOX
Apoptosis rate(%)
si-NC
si-EDEM1
***
**
CON
TG
B
MDA-MB-231
MDA-MB-468
Relative RNA expression
pENTER
Flag-EDEM1
**
ns
sXBP1
ATF4
CHOP
ATF6

Figure S5

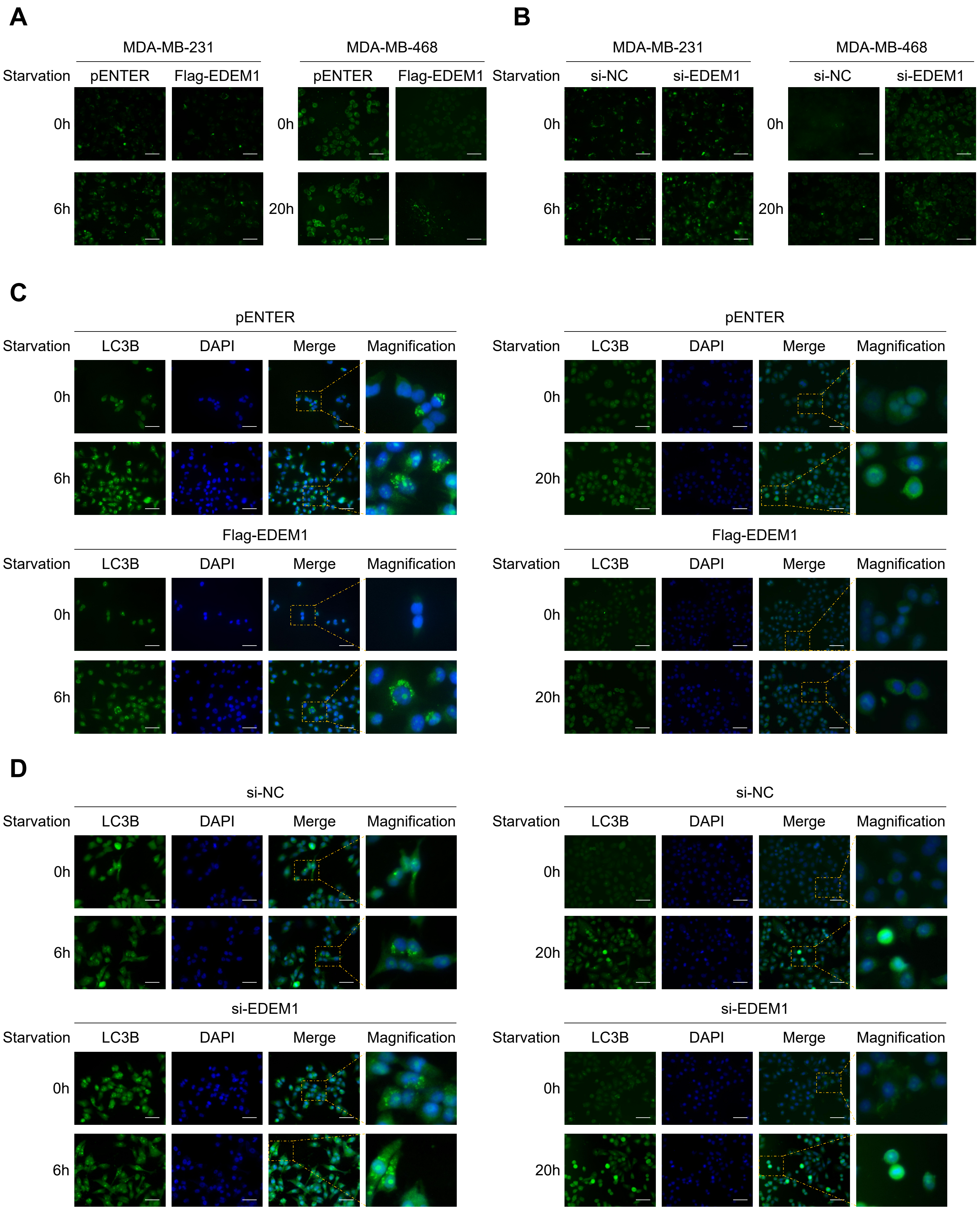
A
MDA-MB-231
MDA-MB-468
Starvation
pENTER
Flag-EDEM1
0h
6h
20h
B
si-NC
si-EDEM1
C
D
LC3B
DAPI
Merge
Magnification

Figure S6

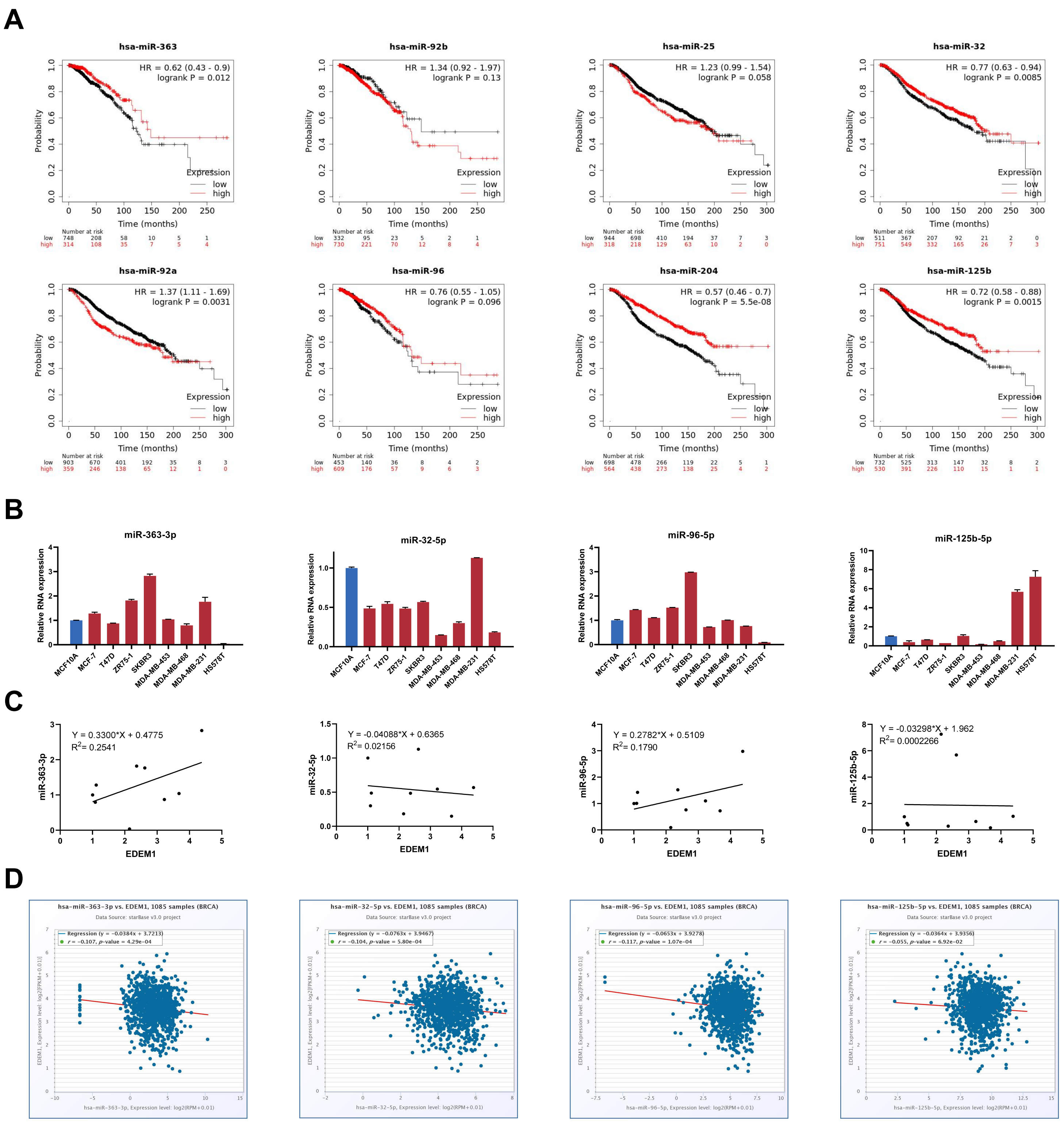
A
hsa-miR-363
HR = 0.62 (0.43 - 0.9)
logrank P = 0.012
hsa-miR-92b
HR = 1.34 (0.92 - 1.97)
logrank P = 0.13
hsa-miR-25
HR = 1.23 (0.99 - 1.54)
logrank P = 0.058
hsa-miR-32
HR = 0.77 (0.63 - 0.94)
logrank P = 0.0085
hsa-miR-92a
HR = 1.37 (1.11 - 1.69)
logrank P = 0.0031
hsa-miR-96
HR = 0.76 (0.55 - 1.05)
logrank P = 0.096
hsa-miR-204
HR = 0.57 (0.46 - 0.7)
logrank P = 5.5e-08
hsa-miR-125b
HR = 0.72 (0.58 - 0.88)
logrank P = 0.0015
Probability
Time (months)
Expression
low
high
Number at risk
B
miR-363-3p
miR-32-5p
miR-96-5p
miR-125b-5p
Relative RNA expression
MCF10A
MCF-7
T47D
ZR75-1
SKBR3
MDA-MB-453
MDA-MB-468
MDA-MB-231
HS578T
C
Y = 0.3300*X + 0.4775
R2= 0.2541
miR-363-3p
Y = -0.04088*X + 0.6365
R2= 0.02156
miR-32-5p
Y = 0.2782*X + 0.5109
R2= 0.1790
miR-96-5p
Y = -0.03298*X + 1.962
R2= 0.0002266
miR-125b-5p
EDEM1
D
hsa-miR-363-3p vs. EDEM1, 1085 samples (BRCA)
hsa-miR-32-5p vs. EDEM1, 1085 samples (BRCA)
hsa-miR-96-5p vs. EDEM1, 1085 samples (BRCA)
hsa-miR-125b-5p vs. EDEM1, 1085 samples (BRCA)
Data Source: starBase v3.0 project

Figure S7

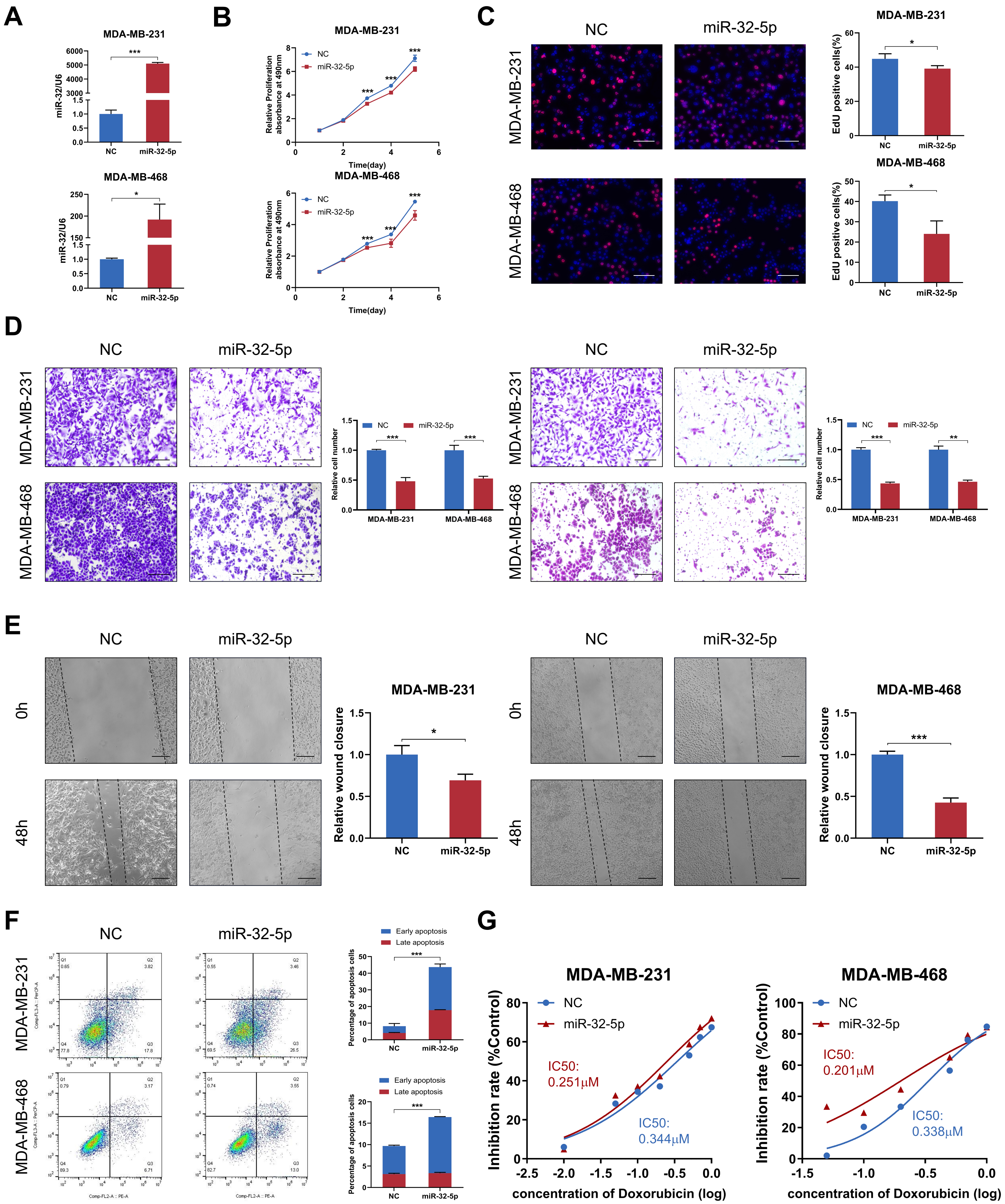
A
MDA-MB-231
miR-32/U6
NC
miR-32-5p
MDA-MB-468
B
Relative Proliferation absorbance at 490nm
Time(day)
C
EdU positive cells(%)
D
Relative cell number
E
0h
48h
Relative wound closure
F
Early apoptosis
Late apoptosis
Percentage of apoptosis cells
G
Inhibition rate (%Control)
concentration of Doxorubicin (log)
IC50: 0.251μM
IC50: 0.344μM
IC50: 0.201μM
IC50: 0.338μM

Figure S8

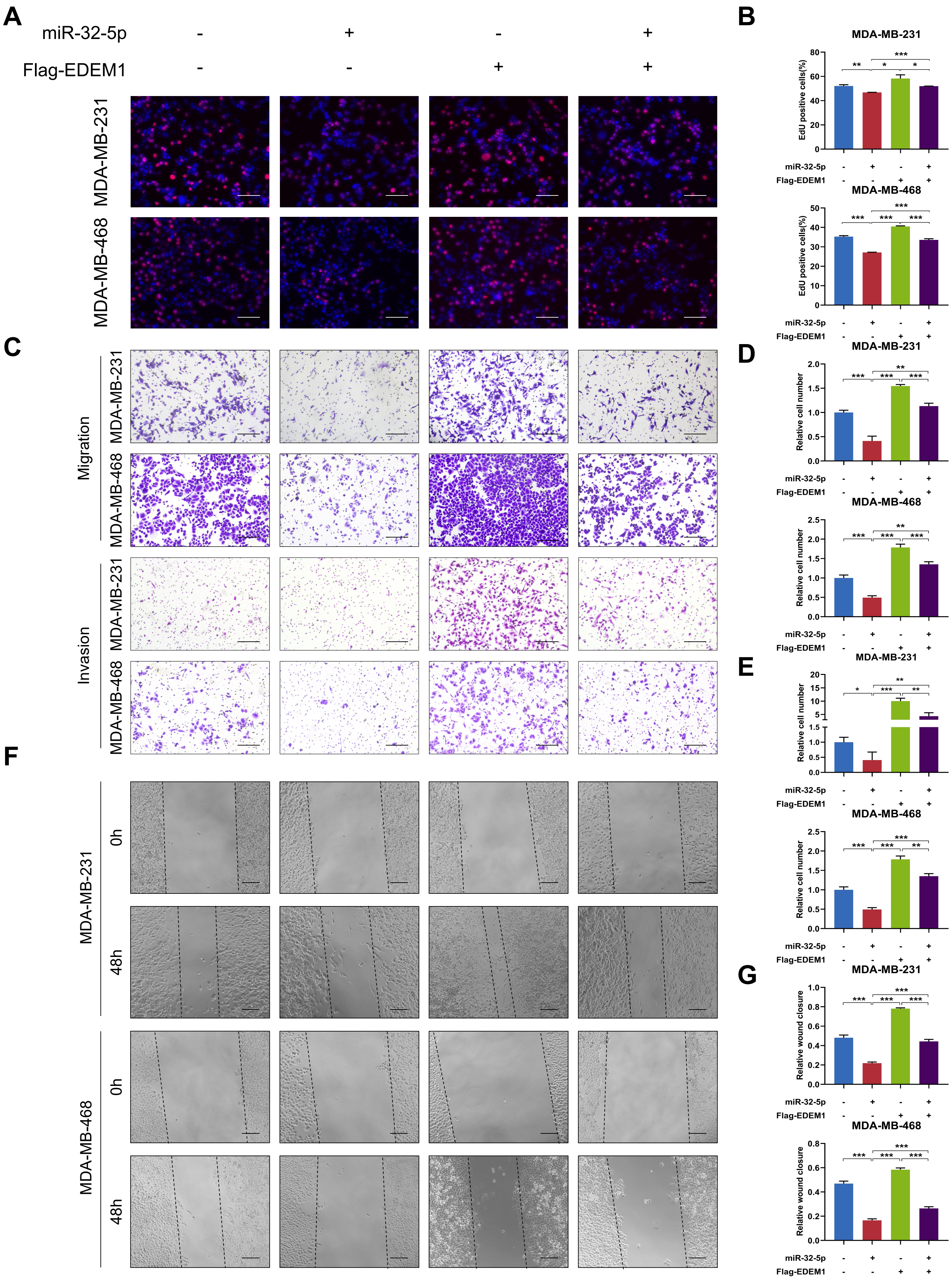

A
miR-32-5p
Flag-EDEM1
MDA-MB-231
MDA-MB-468
B
EdU positive cells(%)
C
Migration
Invasion
D
Relative cell number
E
F
0h
48h
G
Relative wound closure

Figure S9

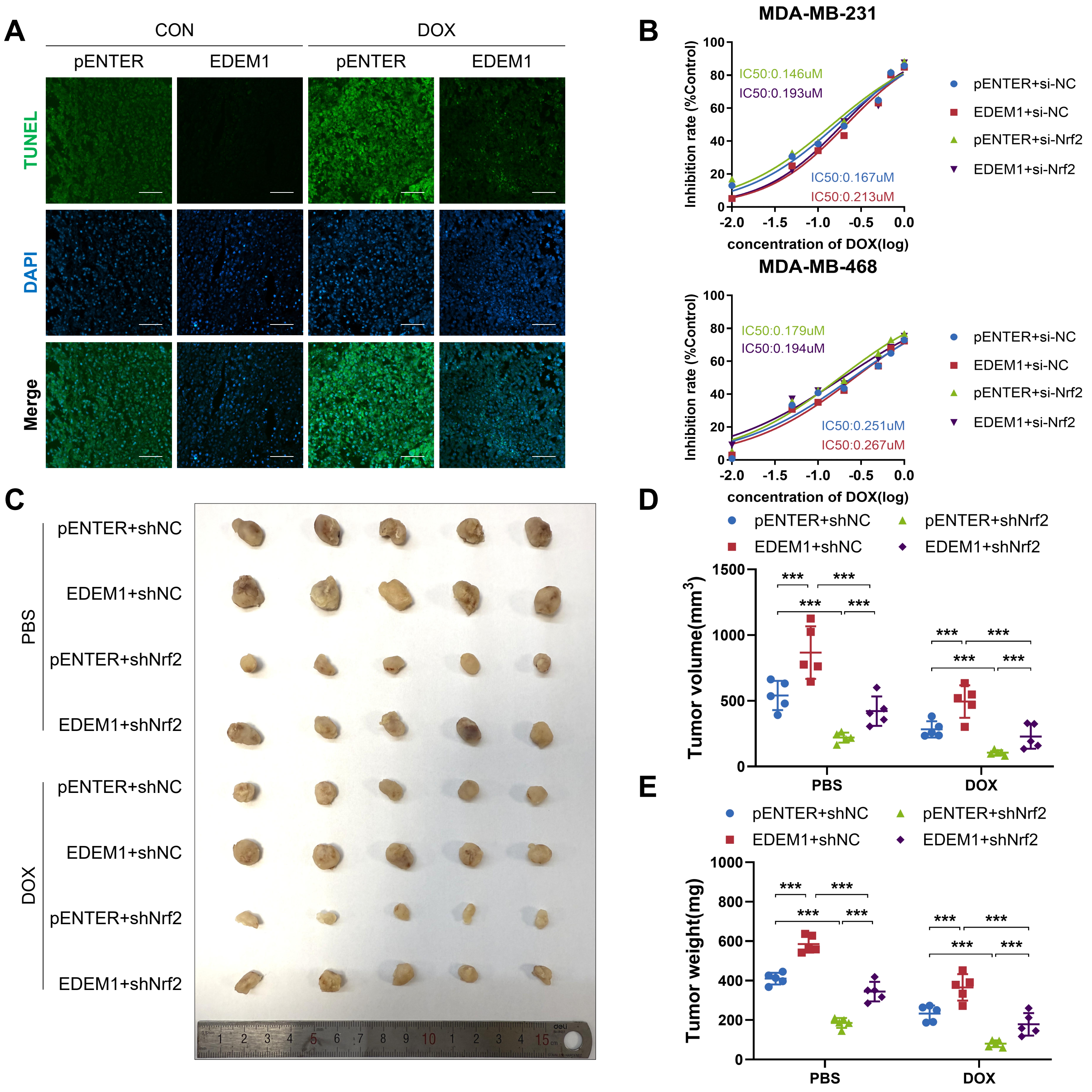

A
CON
DOX
pENTER
EDEM1
TUNEL
DAPI
Merge
B
MDA-MB-231
Inhibition rate (%Control)
IC50:0.146uM
IC50:0.193uM
IC50:0.167uM
IC50:0.213uM
concentration of DOX(log)
pENTER+si-NC
EDEM1+si-NC
pENTER+si-Nrf2
EDEM1+si-Nrf2
MDA-MB-468
IC50:0.179uM
IC50:0.194uM
IC50:0.251uM
IC50:0.267uM
C
PBS
DOX
pENTER+shNC
EDEM1+shNC
pENTER+shNrf2
EDEM1+shNrf2
D
Tumor volume(mm3)
E
Tumor weight(mg)
***

Figure S10

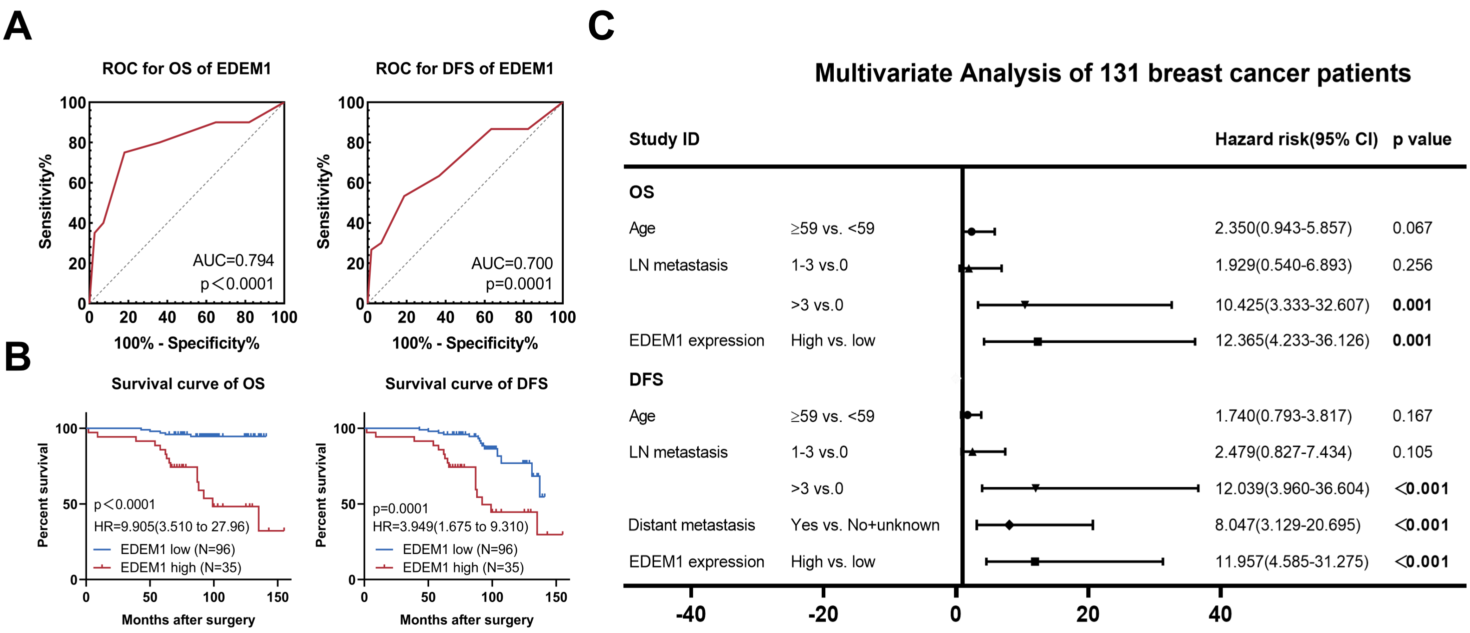

A
ROC for OS of EDEM1
Sensitivity%
100% - Specificity%
AUC=0.794
p<0.0001
ROC for DFS of EDEM1
AUC=0.700
p=0.0001
B
Survival curve of OS
Percent survival
Months after surgery
p<0.0001
HR=9.905(3.510 to 27.96)
EDEM1 low (N=96)
EDEM1 high (N=35)
Survival curve of DFS
p=0.0001
HR=3.949(1.675 to 9.310)
C
Multivariate Analysis of 131 breast cancer patients
Study ID
Hazard risk(95% CI)
p value
OS
Age ≥59 vs. <59 2.350(0.943-5.857) 0.067
LN metastasis 1-3 vs.0 1.929(0.540-6.893) 0.256
>3 vs.0 10.425(3.333-32.607) 0.001
EDEM1 expression High vs. low 12.365(4.233-36.126) 0.001
DFS
Age ≥59 vs. <59 1.740(0.793-3.817) 0.167
LN metastasis 1-3 vs.0 2.479(0.827-7.434) 0.105
>3 vs.0 12.039(3.960-36.604) <0.001
Distant metastasis Yes vs. No+unknown 8.047(3.129-20.695) <0.001
EDEM1 expression High vs. low 11.957(4.585-31.275) <0.001

Supplementary Tables

Table S1. Correlations between EDEM1 expression and clinicopathologic features in 131 breast cancer patients.

Characteristics	EDEM1-low (n=96)	EDEM1-high (n=35)	P value
**Age**			= .237
＜59	73 (76.04 %)	23 (65.71 %)	
≥59	23 (23.96 %)	12 (34.29 %)	
**Tumor stage**			= .390
≤T1	47 (48.96 %)	17 (48.57 %)	
＞T1	49 (51.04 %)	17 (48.57 %)	
Unknown	0 (0.00 %)	1 (2.86 %)	
**LN metastasis**			= .541
0	64 (66.67 %)	20 (57.14 %)	
1-3	17 (17.71 %)	9 (25.71 %)	
＞3	15 (15.62 %)	6 (17.15 %)	
**Distant metastasis**			**＜ .001**
No	79 (82.29 %)	18 (51.43 %)	
Yes	12 (12.50 %)	1 (2.86 %)	
Unknown	5 (5.21 %)	16 (45.71 %)	
**Histologic grade**			= .212
G1	7 (7.29 %)	0 (0.00 %)	
G2	49 (51.04 %)	17 (48.57 %)	
G3	30 (31.25 %)	16 (45.71 %)	
Unknown	10 (10.42 %)	2 (5.72 %)	
**ER status**			**＜ .001**
Negative	46 (47.92 %)	30 (85.71 %)	
Positive	50 (52.08 %)	5 (14.29 %)	
**PR status**			**＜ .001**
Negative	49 (51.04 %)	30 (85.71 %)	
Positive	47 (48.96 %)	5 (14.29 %)	
**HER-2 status**			= .958
Negative	85 (88.54 %)	31 (88.57 %)	
Positive	2 (2.08 %)	1 (2.86 %)	
Unknown	9 (9.38 %)	3 (8.57 %)	
**Ki67 expression**			= .156
Low	24 (25.00 %)	4 (11.43 %)	
High	71 (73.96 %)	30 (85.71 %)	
Unknown	1 (1.04 %)	1 (2.86 %)	

Abbreviation: LN=lymph nodes; ER=estrogen receptor; PR=progesterone receptor; HER-2=human epidermal growth factor receptor-2; P value < .05 marked in bold font to show statistical significance.

Table S2. Univariate and multivariate Cox regression analyses for OS of 131 breast cancer patients.

	Univariate analysis			Multivariate analysis		
	**HR**	**95%CI**	**P value**	**HR**	**95%CI**	**P value**
**Age**						
≥59 vs. ＜59	**3.028**	**1.259-7.281**	**= .013**	2.350	0.943-5.857	= .067
**Histologic grade**						
G2 vs. G1	9317.972	0.000-3.001E+78	= .917	-	-	-
G3 vs. G1	8650.070	0.000-2.787E+78	= .918	-	-	-
Unknown vs. G1	8523.247	0.000-2.758E+78	= .918	-	-	-
**Tumor size**						
＞T1 vs.≤T1+Unknown	1.313	0.543-3.172	= .545	-	-	-
**LN metastasis**						
1-3 vs. 0	1.983	0.580-6.780	= .275	1.929	0.540-6.893	= .256
＞3 vs. 0	**8.050**	**2.902-22.326**	**＜.001**	**10.425**	**3.333-32.607**	**= .001**
**Distant metastasis**						
Yes vs. No+Unknown	1.579	0.462-5.394	= .466	-	-	-
**ER status**						
Pos vs. Neg	1.017	0.400-2.584	= .973	-	-	-
**PR status**						
Pos vs. Neg	0.878	0.339-2.272	= .788	-	-	-
**HER-2 status**						
Pos vs. Neg+Unknown	2.433	0.322-18.388	= .389	-	-	-
**Ki67 expression**						
High vs. Low+Unknown	1.185	0.395-3.559	= .762	-	-	-
**EDEM1 expression**						
High vs. Low	**10.230**	**3.694-28.330**	**＜.001**	**12.365**	**4.233-36.126**	**= .001**

Abbreviation: 95% CI=95% confidence interval; HR=hazard ratio; LN=lymph nodes; ER=estrogen receptor; PR=progesterone receptor; HER-2: human epidermal growth factor receptor-2; P value < .05 marked in bold font to show statistical significance.

Table S3. Univariate and multivariate Cox regression analyses for DFS of 131 breast cancer patients.

	Univariate analysis			Multivariate analysis		
	**HR**	**95%CI**	**P value**	**HR**	**95%CI**	**P value**
**Age**						
≥59 vs. ＜59	**2.412**	**1.169-4.975**	**= .017**	1.740	0.793-3.817	= .167
**Histologic grade**						
G2 vs. G1	9683.222	0.000-1.018E+69	= .904	-	-	-
G3 vs. G1	9420.873	0.000-9.911E+68	= .905	-	-	-
Unknown vs. G1	5068.560	0.000-5.361E+68	= .911	-	-	-
**Tumor size**						
＞T1 vs. ≤T1+Unknown	1.638	(0.788-3.407)	= .187	-	-	-
**LN metastasis**						
1-3 vs. 0	**2.956**	**1.162-7.520**	**= .023**	2.479	0.827-7.434	= .105
＞3 vs. 0	**10.279**	**4.180-25.280**	**＜ .001**	**12.039**	**3.960-36.604**	**＜ .001**
**Distant metastasis**						
Yes vs. No+Unknown	**6.412**	**3.105-13.241**	**＜ .001**	**8.047**	**3.129-20.695**	**＜ .001**
**ER status**						
Pos vs. Neg	1.531	0.683-3.432	= .301	-	-	-
**PR status**						
Pos vs. Neg	1.396	0.625-3.120	= .415	-	-	-
**HER-2 status**						
Pos vs. Neg+Unknown	2.242	0.298-16.885	= .433	-	-	-
**Ki67 expression**						
Pos vs. Neg+Unknown)	0.932	0.398-2.183	= .871	-	-	-
**EDEM1 expression**						
High vs. Low	**4.053**	**1.963-8.370**	**＜ .001**	**11.957**	**4.585-31.275**	**＜ .001**

Abbreviation: 95% CI=95% confidence interval; HR=hazard ratio; LN=lymph nodes; ER=estrogen receptor; PR=progesterone receptor; HER-2: human epidermal growth factor receptor-2; p value < .05 marked in bold font to show statistical significance.

Table S4. Correlations between EDEM1 expression and clinicopathologic features in 76 TNBC patients.

Characteristics	EDEM1-low(n=46)	EDEM1-high(n=30)	P value
**Age**			= .968
＜59	32 (69.57 %)	21 (70.00 %)	
≥59	14 (30.43 %)	9 (30.00 %)	
**Tumor stage**			= .529
≤T1	20 (43.48 %)	14 (46.67 %)	
＞T1	26 (56.52 %)	15 (50.00 %)	
Unknown	0 (0.00 %)	1 (3.33 %)	
**LN metastasis**			= .531
0	32 (69.57 %)	18 (60.00 %)	
1-3	10 (21.74 %)	7 (23.33 %)	
＞3	4 (8.69 %)	5 (16.67 %)	
**Distant metastasis**			**＜ .001**
No	40 (86.96 %)	17 (56.67 %)	
Yes	6 (13.04 %)	0 (0.00 %)	
Unknown	0 (0.00 %)	13 (43.33 %)	
**Histologic grade**			= .557
G1	0 (0.00 %)	0 (0.00 %)	
G2	21 (45.65 %)	14 (46.67 %)	
G3	20 (43.48 %)	15 (50.00 %)	
Unknown	5 (10.87 %)	1 (3.33 %)	
**Ki67 expression**			= .679
Low	6 (13.04 %)	3 (10.00 %)	
High	40 (86.96 %)	26 (86.67 %)	
Unknown	0 (0.00 %)	1 (3.33 %)	

Abbreviation: LN=lymph nodes; P value < 0.05 marked in bold font to show statistical significance.

Table S5. Univariate and multivariate Cox regression analyses for OS of 76 TNBC patients.

	Univariate analysis			Multivariate analysis		
	**HR**	**95%CI**	**P value**	**HR**	**95%CI**	**P value**
**Age**						
≥59 vs. ＜59	2.454	0.791-7.618	= .120	-	-	-
**Histologic grade**						
G3 vs. G2	0.868	0.264-2.851	= .816	-	-	-
Unknown vs. G2	0.734	0.088-6.154	= .776	-	-	-
**Tumor size**						
＞T1 vs.≤T1+Unknown	3.173	0.856-11.767	= .084	-	-	-
**LN metastasis**						
1-3 vs. 0	2.515	0.562-11.256	= .228	4.152	0.832-20.715	= .083
＞3 vs. 0	**14.310**	**3.564-57.453**	**＜.001**	**19.072**	**3.646-99.771**	**= .001**
**Distant metastasis**						
Yes vs. No+Unknown	0.965	0.124-7.508	= .973	-	-	-
**Ki67 expression**						
High vs. Low+Unknown	0.464	0.123-1.751	= .257	-	-	-
**EDEM1 expression**						
High vs. Low	**21.181**	**2.727-164.538**	**= .004**	**25.659**	**3.107-211.906**	**= .003**

Abbreviation: 95% CI=95% confidence interval; HR=hazard ratio; LN=lymph nodes; P value < 0.05 marked in bold font to show statistical significance.

Table S6. Univariate and multivariate Cox regression analyses for DFS of 76 TNBC patients.

	Univariate analysis			Multivariate analysis		
	**HR**	**95% CI**	**P value**	**HR**	**95% CI**	**P value**
**Age**						
≥59 vs. ＜59	2.393	0.917-6.245	= .075	-	-	-
**Histologic grade**						
G3 vs. G2	0.901	0.334-2.434	= .838	-	-	-
Unknown vs. G2	0.404	0.050-3.234	= .393	-	-	-
**Tumor size**						
＞T1 vs. ≤T1+Unknown	2.651	0.929-7.565	= .069	-	-	-
**LN metastasis**						
1-3 vs. 0	2.968	0.899-9.798	= .074	**6.739**	**1.426-31.843**	**= .016**
＞3 vs. 0	**19.353**	**5.280-70.935**	**＜.001**	**28.044**	**4.877-161.250**	**＜.001**
**Distant metastasis**						
Yes vs. No+unknown	**4.888**	**1.784-13.389**	**= .002**	188288.696	0.000-5.938E+77	= .887
**Ki67 expression**						
High vs. Low+Unknown	0.553	0.176-1.737	= .310	-	-	-
**EDEM1 expression**						
High vs. Low	**3.852**	**1.417-10.472**	**= .008**	223708.333	0.000-7.057E+77	= .885

Abbreviation: 95% CI=95% confidence interval; HR=hazard ratio; LN=lymph nodes; P value < 0.05 marked in bold font to show statistical significance.

Table S7. SiRNAs and mimics for transfection.

**Name**	**Sequence （5’-3’）**
si-EDEM1	CCCUCCCUGAGAGAUAUAATTUUAUAUCUCUCAGGGAGGGTT
si-NC	UUCUCCGAACGUGUCACGUACGUGACACGUUCGGAGAA
hsa-miR-32-5p	UAUUGCACAUUACUAAGUUGCA

Table S8. Primers used for qRT-PCR.

Name	Forward (5'-3')	Reverse (5'-3')
β-actin	CACCATTGGCAATGAGCGGTTC	AGGTCTTTGCGGATGTCCACGT
EDEM1	GGGTTGGAAAGCAGAGTG	GTTGACATAGAGTGGAGGGT
EDEM2	CAACATTCCTCAGGGATACAC	TTCCGAGTTCTAGGAGGGT
EDEM3	ACACTGGGCTCAACATCC	AAATCCGCAAGGCACTCTA
SOD1	GGGCATCATCAATTTCGA	AGCCTGCTGTATTATCTCC
SOD2	CGACCTGCCCTACGACTA	AACGCCTCCTGGTACTTCT
NQO1	GAGTGGCATTCTGCATTT	TATTCTCCAGGCGTTTCT
HO-1	CAGCGGGCCAGCAACAAA	ACCCATCGGAGAAGCGGAG
